# Most “Dark Matter” Transcripts Are Associated With Known Genes

**DOI:** 10.1371/journal.pbio.1000371

**Published:** 2010-05-18

**Authors:** Harm van Bakel, Corey Nislow, Benjamin J. Blencowe, Timothy R. Hughes

**Affiliations:** 1 Banting and Best Department of Medical Research, University of Toronto, Toronto, Ontario, Canada; 2 Department of Molecular Genetics, University of Toronto, Toronto, Ontario, Canada; HHMI Janelia Farm, United States of America

## Abstract

A series of reports over the last few years have indicated that a much larger portion of the mammalian genome is transcribed than can be accounted for by currently annotated genes, but the quantity and nature of these additional transcripts remains unclear. Here, we have used data from single- and paired-end RNA-Seq and tiling arrays to assess the quantity and composition of transcripts in PolyA+ RNA from human and mouse tissues. Relative to tiling arrays, RNA-Seq identifies many fewer transcribed regions (“seqfrags”) outside known exons and ncRNAs. Most nonexonic seqfrags are in introns, raising the possibility that they are fragments of pre-mRNAs. The chromosomal locations of the majority of intergenic seqfrags in RNA-Seq data are near known genes, consistent with alternative cleavage and polyadenylation site usage, promoter- and terminator-associated transcripts, or new alternative exons; indeed, reads that bridge splice sites identified 4,544 new exons, affecting 3,554 genes. Most of the remaining seqfrags correspond to either single reads that display characteristics of random sampling from a low-level background or several thousand small transcripts (median length = 111 bp) present at higher levels, which also tend to display sequence conservation and originate from regions with open chromatin. We conclude that, while there are bona fide new intergenic transcripts, their number and abundance is generally low in comparison to known exons, and the genome is not as pervasively transcribed as previously reported.

## Abstract 2

Short-read RNA sequencing in mouse and human tissues shows that most transcripts are encoded within or nearby known genes and that most of the genome is not transcribed.

## Author Summary

The human genome was sequenced a decade ago, but its exact gene composition remains a subject of debate. The number of protein-coding genes is much lower than initially expected, and the number of distinct transcripts is much larger than the number of protein-coding genes. Moreover, the proportion of the genome that is transcribed in any given cell type remains an open question: results from “tiling” microarray analyses suggest that transcription is pervasive and that most of the genome is transcribed, whereas new deep sequencing-based methods suggest that most transcripts originate from known genes. We have addressed this discrepancy by comparing samples from the same tissues using both technologies. Our analyses indicate that RNA sequencing appears more reliable for transcripts with low expression levels, that most transcripts correspond to known genes or are near known genes, and that many transcripts may represent new exons or aberrant products of the transcription process. We also identify several thousand small transcripts that map outside known genes; their sequences are often conserved and are often encoded in regions of open chromatin. We propose that most of these transcripts may be by-products of the activity of enhancers, which associate with promoters as part of their role as long-range gene regulatory sites. Overall, however, we find that most of the genome is not appreciably transcribed.

## Introduction

In recent years established views of transcription have been challenged by the observation that a much larger portion of the human and mouse genomes is transcribed than can be accounted for by currently annotated coding and noncoding genes. The bulk of these findings have come from experiments using “tiling” microarrays with probes that cover the non-repetitive genome at regular intervals [1]–[9], or from sequencing efforts of full-length cDNA libraries enriched for rare transcripts [10],[11]. Additionally, capped analysis of gene expression (CAGE) in human and mouse show that a significant number of sequenced 5′ tags map to intergenic regions [12]. Estimates of the proportion of transcripts that map to locations separate from known exons range from 47% to 80% and are distributed approximately equally between introns and intergenic regions. Dubbed transcriptional “dark matter” [13], the “hidden” transcriptome [1], or transcripts of unknown function (TUFs) [4],[14], the exact nature of much of this additional transcription is unclear, but it has been presumed to comprise a combination of novel protein coding transcripts, extensions of existing transcripts, noncoding RNAs (ncRNAs), antisense transcripts, and biological or experimental background. Determining the relative contributions of each of these potential sources is important for understanding the nature and possible biological function of transcriptional dark matter.

Homology searches for transcripts mapping outside known annotation boundaries [10], as well as cDNA sequencing efforts, indicate that it is still possible to find new exons of protein coding genes [10],[15],[16]. The genomic positions of TUFs are also biased towards known transcripts [8], suggesting that at least a portion may represent extensions of current gene annotations. Nevertheless, the majority of dark matter transcripts is thought to be noncoding [2],[4],[5],[10]. Previous efforts to characterize dark matter transcripts have revealed the existence of thousands of ncRNAs with evidence for tissue-specific expression [17],[18], as well as over a thousand large intervening noncoding RNAs (lincRNAs) originating from intergenic regions bearing chromatin marks associated with transcription [19]. Other studies have reported new classes of ncRNAs, such as those that cluster close to the transcription start sites (TSSs) of protein coding genes [20]–[24]. These promoter-associated RNAs (pasRNAs) typically initiate in the nucleosome free regions that mark a TSS, with transcription occurring in both directions. Finally, results from the ENCODE pilot project have suggested a highly interleaved structure of the human transcriptome, with an estimate that as much as 93% of the human genome may give rise to primary transcripts [9]. Though this estimate was based on a combination of sources that included rapid amplification of cDNA ends coupled to detection on tiling arrays (RACE-tiling), manually curated GENCODE annotations, and paired-end sequencing of long cDNAs (GIS-PET), it was dominated by the results of RACE-tiling experiments that alone found 80% genome coverage, compared to 64.6% and 66.4% for GENCODE annotations and GIS-PET, respectively.

The fact that most TUFs do not appear to be under evolutionary selective pressure [25] has prompted suggestions that at least some of the transcriptional dark matter may constitute “leaky” background transcription [9],[26]. Consistent with this notion, many of the intergenic and intronic transcripts are detected at low levels, close to the detection limit of qPCR or Northern blots [13]. Presumably as a consequence, validation rates for unannotated transcribed regions detected in tiling array experiments have varied between 25% and 70% [1],[5],[27], and a comparison [13] of human chromosome 22 data from three major tiling array studies done on different platforms [1],[3],[27] also revealed little overlap of expressed probes, with 89% of overlapping positive probes mapping to exons or introns of known transcripts. While this low overlap may be due to differences in the samples analyzed [4], there is also evidence that some dark matter transcripts may be due to experimental artifacts. For example, a reassessment of the analysis parameters used in the tiling array study by Kampa et al. [2] revealed a similar number of transcribed fragments in real and randomized microarray data [28]. These issues make it difficult to assess the level of false positives in tiling array experiments.

Transcriptome sequencing (RNA-Seq) has emerged as a new technology that does not suffer from many of the limitations of array platforms such as cross-hybridization [29]. The technique has a wide dynamic range spanning at least four to five orders of magnitude [30],[31] and allows accurate quantitation of expression levels, as determined by experiments using externally spiked-in RNA controls and quantitative PCR [30]. These characteristics make RNA-Seq suitable to accurately assess the relative proportion of sequence from the known versus the dark matter transcriptome. Comparisons between studies of eukaryotic transcriptomes have shown that the estimated proportion of transcriptional dark matter reported in RNA-Seq studies is consistently lower than estimates from tiling arrays [32]. Although most RNA-Seq studies to date have focused on polyadenylated (PolyA+) RNA, which would be enriched for coding transcripts, this cannot fully account for the differences, as most tiling array studies show nearly the same degree of nonexonic transcription for PolyA+ as for total RNA sources [1]–[9]. Indeed, it was reported that even in the most mature form of PolyA+ RNA isolated from the cytosol, approximately half of the transcribed sequence does not correspond to known exons [5]. Moreover, RNA-Seq data from Arabidobsis rRNA-depleted total RNA samples contained a relatively small proportion (3.5%) of intergenic reads [33]. These results may not be characteristic of the larger and more complex human and mouse transcriptomes, but they do present an example in which the proportion of dark matter transcripts is relatively low in a more heterogeneous RNA pool. Other studies, in contrast, reported a higher proportion of nonexonic reads in yeast [34] and for total RNA in human [35], leaving unresolved the question of the quantity and character of dark matter transcripts.

To investigate the extent and nature of transcriptional dark matter, we have analyzed a diverse set of human and mouse tissues and cell lines using tiling microarrays and RNA-Seq. A meta-analysis of single- and paired-end read RNA-Seq data reveals that the proportion of transcripts originating from intergenic and intronic regions is much lower than identified by whole-genome tiling arrays, which appear to suffer from high false-positive rates for transcripts expressed at low levels. The majority of RNA-Seq reads that map to intergenic regions either display a high degree of correlation with neighboring genes or are associated with more than 10,000 potential novel exonic fragments we identified in human and mouse. A genome-wide analysis of “*de novo*” splice junctions in human samples further revealed 2,789 previously uncharacterized transcript fragments that have no overlap with exons of known gene annotations, 1,259 of which map to intergenic regions. We also find 4,544 additional exons for annotated transcripts, 723 of which extend transcripts at the 5′ end and include likely alternative promoters. The novel exons from spliced transcripts are supported by EST data, are generally more conserved, and derive from coding as well as noncoding transcripts. We conclude that analysis of data from tiling arrays leads to vast overestimates of the proportion of transcriptional dark matter. However, the mammalian transcriptome does contain thousands of unannotated transcripts, exons, promoters, and termination sites. Intriguingly, there is a strong overlap of short intergenic transcripts with DNase I hypersensitive sites, suggesting that they may be the equivalent of pasRNAs for distant enhancers.

## Results

### High False-Positive Rate from Tiling Arrays

We directly compared the accuracy of tiling arrays and RNA-Seq in identifying known transcribed regions from polyadenylated (PolyA+) RNA. To avoid potential genomic abnormalities of cell lines we mainly focused on transcriptome data from tissue sources. For microarray expression profiling, we used Affymetrix whole-genome tiling arrays at a 35 bp resolution for four human and four mouse tissues. In addition, we generated RNA-Seq data for cDNA fragments from human whole brain tissue (multiple donors) and a mixture of cell lines, which were sequenced at both ends on an Illumina genome analyzer to an average depth of 23 M paired 50 nt reads per sample. To match coverage across a wider variety of tissues, we supplemented the paired-end RNA-Seq data with publicly available 32 nt single-end PolyA+ selected datasets, sequenced to an average depth of 22 M reads for 8 human tissues from single donors [16]. RNA-Seq data for mouse were obtained from Mortazavi et al. [36] and consisted of 25 nt single-end data for PolyA+ RNA from three tissues, sequenced to an average depth of 73 M reads. The resulting combined dataset contained tissue-matched RNA-Seq and tiling array data for 4 human and 3 mouse tissues. For our analyses, we only considered RNA-Seq reads that could be unequivocally mapped to unique positions in the genome. This avoided erroneous identification of transcribed regions and facilitated comparisons to data obtained from tiling arrays, which were designed for the non-repetitive part of the genome. Overall the total number of uniquely mapped reads numbered 185.6 M and 79.8 M for the human and mouse genomes, respectively (see Table S1 for a breakdown per tissue). Since the arrays contained only perfect-match probes, the raw intensity data were normalized against a genomic DNA reference to correct for any bias in probe sequence composition (Materials and Methods).

**Figure 1:**
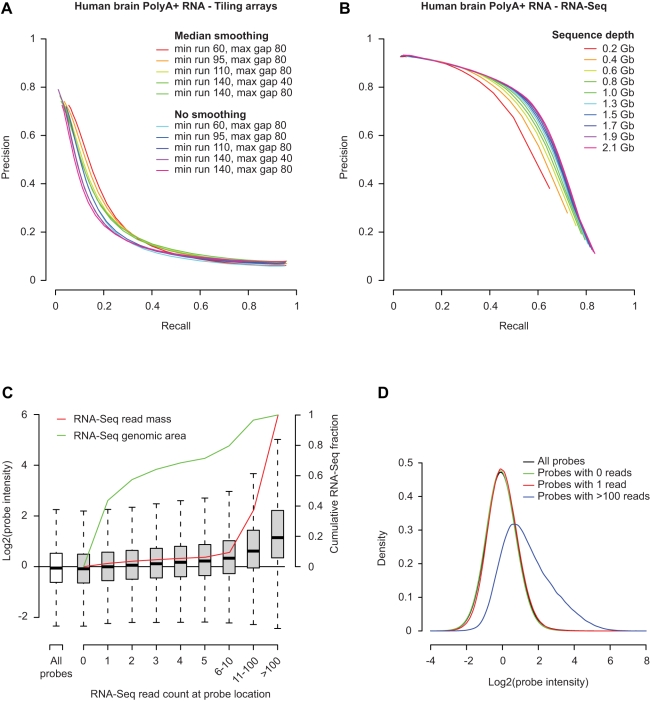
Low precision for tiling arrays compared to RNA-Seq data. (A) Precision-recall curves for detection of exons in human RefSeq gene annotations on tiling arrays. Transcribed genomic regions (transfrags) were selected based on a range of parameters that were applied before or after median smoothing with a bandwidth of 70 bp: max gap, the maximum distance between two positive probes; min run, the minimum size of a transcribed region. The log2 normalized intensity threshold used to select positive probes was varied between −1 and 2 to plot each line. (B) Precision-recall curves for the combined RNA-Seq data from three human brain samples, at different read depths (0.2 to 2.1 Gb). Transcribed regions (seqfrags) were identified on the basis of uniquely mapped reads, and the threshold for the minimal read count per seqfrag was varied between 1 and 100 to plot each line. (C) Comparison of RNA-Seq read counts and tiling array probe intensities for the pooled set of human brain RNA-Seq reads (three samples). The number of RNA-Seq reads overlapping each mapped probe coordinate was determined and used to draw a boxplot of the intensity distributions measured for probes overlapped by varying numbers of RNA-Seq reads, as indicated (gray boxes). The intensity distribution across all probes is shown in comparison (white box). Line graphs indicating the cumulative fraction of RNA-Seq read area (green) and read count (red) covered at each read coverage level are superimposed on the barplot, with the scale shown on the right. (D) Kernel-density plot of probe intensities for high- and low-coverage probe groups from (A), as indicated.

We compared the performance of tiling arrays and RNA-Seq for human total brain tissue, since it had the highest combined sequence coverage of any tissue used in this study (50.2 M uniquely mapped reads from three independent samples, corresponding to 2.1 Gb of sequencing data). Figures 1A and 1B show the relation between the fraction of detected transcript fragments on tiling arrays (transfrags) or in RNA-Seq data (seqfrags) that overlap known RefSeq exons (i.e., precision) and the total fraction of exons recovered (i.e., recall). Tiling array transfrags were identified by selecting consecutive probes that scored above a range of intensity thresholds, with additional limits on the minimum length of each transfrag (minrun) and the maximum gap between probes meeting the threshold (maxgap). The analysis was performed directly on the normalized intensity data, or after applying additional median smoothing across neighboring probes in the genome within a sliding window, to reduce intensity variability. Seqfrags were defined as consecutively transcribed regions in the uniquely mapped RNA-Seq data, and performance was evaluated over a range of thresholds set on the minimum number of reads per seqfrag. We find that RNA-Seq offers superior precision in identifying RefSeq exons compared to tiling arrays, while achieving a high level of recall (Figure 1A, 1B). This difference remains apparent even over a broad range of parameter settings typically used to identify transcribed regions in tiling array data. These observations do not directly demonstrate that tiling arrays have a higher false-positive rate, as a lower precision would also be expected if the majority of the genome were transcribed: the difference between platforms could also reflect a lack of sensitivity to detect unannotated transcripts expressed at lower levels in RNA-Seq data, due to insufficient sequencing depth. If this were the case, however, we would expect that the precision-recall curves for RNA-Seq data would look progressively more similar to those of the tiling arrays with increasing read counts. Instead, when we examined the effect of varying sequencing depth by sampling smaller subsets of reads from the combined human brain RNA-Seq datasets we found that increased sequencing improves recall without a loss in precision (Figure 1B). Thus, the discrepancy with tiling arrays increases rather than decreases with greater sequencing depths.

We also directly compared RNA-Seq read coverage with tiling array measurements at the same genomic location. Figure 1C shows a direct comparison between the number of reads and the normalized probe signal intensity. Consistent with the precision-recall curves that show that high precision in tiling array experiments is only achieved at the most stringent intensity thresholds (Figure 1A), we find that the agreement between sequencing data and array intensities data is poor for all but the most highly transcribed regions. Indeed, the normalized intensity distribution for tiling array probes overlapping transcribed regions in RNA-Seq data with single-read coverage is essentially random (Figure 1D), consistent with previous observations that the correlation between RNA-Seq data and tiling arrays is poor for transcripts expressed at low levels [29],[36]. We do note, however, that the tiling arrays and RNA-Seq data generally agree on the location of the greatest transcript mass (Figure 1C, red line). The increased precision of RNA-Seq is presumably due to reduced ambiguity in detecting transcripts at lower expression levels, relative to microarrays, in which signal from cross-hybridization increasingly contributes to false-positive detection at low expression levels. It is thus conceivable that the proportion of dark matter transcripts based on tiling array experiments is considerably overestimated. Given the improved performance of RNA-Seq over tiling arrays, we therefore focused on RNA-Seq data to revisit the nature of dark matter transcripts.

### Dark Matter Transcripts Make up a Small Fraction of the Total Sequenced Transcript Mass

**Figure 2:**
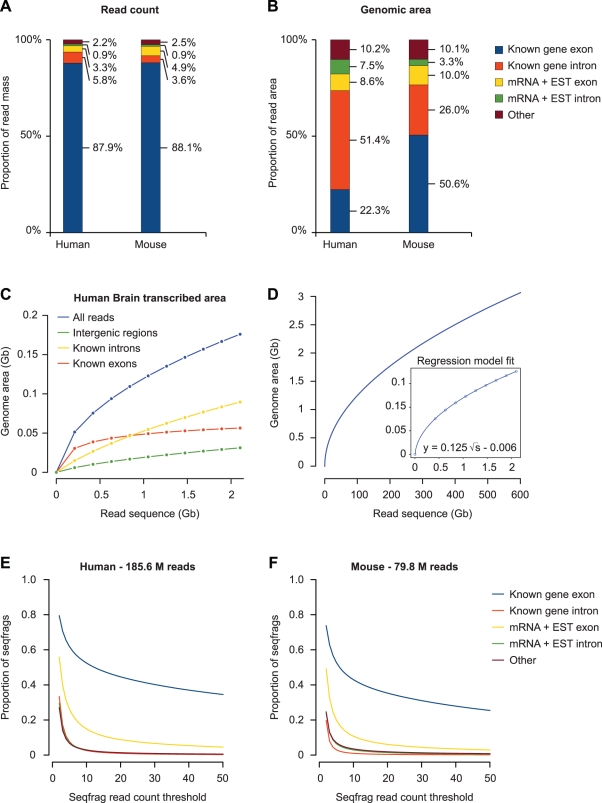
RNA-Seq read mapping overview. (A) Proportion of reads with a unique match in the genome mapping to known genes, mRNAs, and spliced ESTs. Reads were pooled across all human or mouse RNA-Seq samples and sequentially matched against a non-redundant set of known genes, mRNA, and spliced EST data. Any remaining reads were classified as “other.” (B) Same as in (A) but considering the total amount of transcribed genomic area, rather than read count. (C) The relationship between the RNA-Seq read depth and the transcribed area in the genome for human brain RNA-Seq reads, based on 50.2 million reads pooled from the three independent samples that were assayed separately. The total transcribed area is indicated for all reads, as well as those that map to known exons, known introns, and intergenic regions. (D) Extrapolation of transcribed genomic area at increasing read depths, based on the distribution of all reads in (C). The model fitted on the uniquely mapped reads is shown in the inset. (E, F) Cumulative fraction of seqfrags as a function of the number of reads mapped to each seqfrags in the combined set of human and mouse samples, respectively.

To assess the proportion of unique sequence-mapping reads accounted for by dark matter transcripts in RNA-Seq data, we compared the mapped sequencing data to the combined set of known gene annotations from the three major genome databases (UCSC, NCBI, and ENSEMBL, together referred to here as “annotated” or “known” genes). When considering uniquely mapped reads in all human and mouse samples, the vast majority of reads (88%) originate from exonic regions of known genes (Figure 2A). These figures are consistent with previously reported fractions of exonic reads of between 75% and 96% for unique reads [16],[33],[36]–[38], including those of the original studies from which some of the RNA-Seq data in this study were derived. When including introns, as much as 92%–93% of all reads can be accounted for by annotated gene regions. A further 4%–5% of reads map to unannotated genomic regions that can be aligned to spliced ESTs and mRNAs from high-throughput cDNA sequencing efforts, and only 2.2%–2.5% of reads cannot be explained by any of the aforementioned categories. The proportions of mapped reads are consistent between tissues and cell lines and independent of read sequence length (Table S1). Altogether, dark matter transcripts only account for a small proportion of PolyA+ transcripts.

While annotated exons can explain the majority of reads, they make up a much smaller proportion of the total transcribed area of the genome: 22.3% in human and 50.6% in mouse (Figure 2B). Nevertheless, complete annotated gene structures in both organisms still account for ∼75% of the total transcribed area. The apparent discrepancy in transcribed intronic versus exonic area in human versus mouse is directly related to the combined increased sequencing depth for the human samples (Table S2). This is illustrated in Figure 2C, which shows the relationship between the amount of sequence coverage in the combined PolyA+ RNA-Seq data from human brain samples and the transcribed area. While the exonic transcribed area levels off quickly at around 500 Mb of RNA-Seq coverage, intergenic and intronic areas keep increasing at roughly constant rates. When we extrapolate from the observed relationship between the amount of mapped sequence data and genomic area covered (Figure 2D), we find that given sufficient sequencing depth the whole genome may appear as transcripts. However, the fact that such pervasive transcription would only be detected at sequencing depths more than two orders of magnitude above current levels suggests that these transcripts may largely be attributed to biological and/or technical background. Indeed, the vast majority of intergenic and intronic seqfrags have very low sequence coverage (Figure 2E, 2F), exemplified by the fact that 70% (human) to 80% (mouse) of the transcribed area in these regions is detected by a single RNA-Seq read in only one sample, much of which is consistent with random placement (see below).

The low coverage and ubiquitous character of the intronic seqfrags suggests that they may represent random sampling from partially processed or unprocessed RNAs. We also note that 4.5% of all mapped (non-unique) human RNA-Seq reads correspond to rRNAs and sn(o)RNAs, suggesting that the PolyA+ selection did not fully exclude RNAs that are not polyadenylated. Alternatively, some of these transcripts may be polyadenylated under normal conditions, or they could correspond to degradation intermediates [39]. We note that, as the number of reads increases, the amount of transcribed area in intergenic regions increases at a much lower rate than in intronic regions (Figure 2C), even though intergenic regions make up a larger proportion of the human genome (1.7 Gb compared to 1.3 Gb for introns), further supporting the notion of random sampling of introns. In the complete set of uniquely mapped human brain RNA-Seq data, intergenic reads appear 3.8-fold less often than reads in intronic regions. In contrast, the cumulative read coverage is much higher for mRNA and EST exons than it is for either introns or intergenic regions (Figure 2E, 2F), indicating that many mRNAs and ESTs likely constitute valid transcripts that are not currently annotated in the three major genome databases. In summary, even though the genome may be randomly transcribed at very low levels, the vast majority of sequence reads in PolyA+ samples corresponds to known genes and transcripts, arguing against widespread transcription to the extent reported previously.

### Most Intergenic Transcripts Are Adjacent to Known Genes

**Figure 3:**
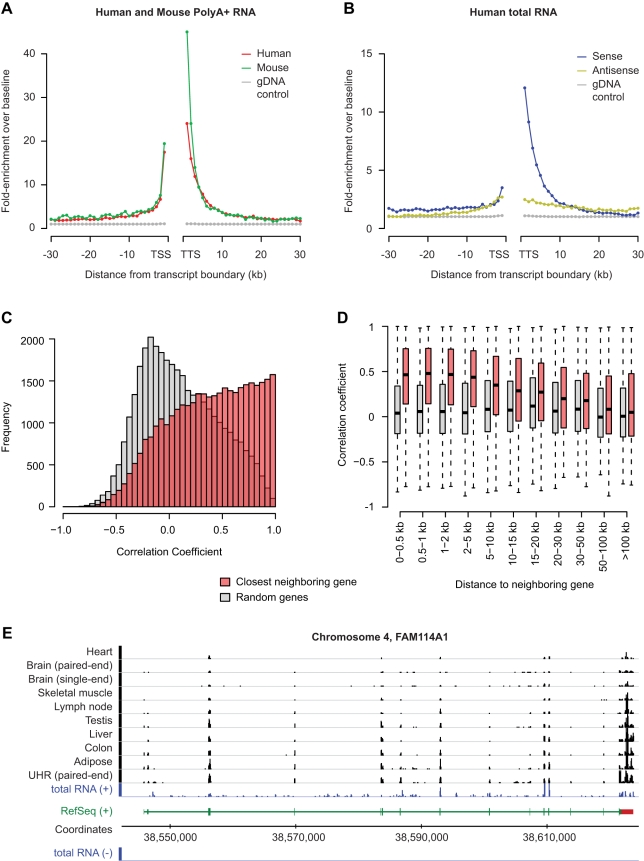
Intergenic expression is positionally biased towards known genes. (A) Relative enrichment of RNA-Seq read frequency in intergenic regions as a function of the distance to 5′ and 3′ ends of annotated genes in the human (red) and mouse genomes (green). The distribution in genomic DNA-Seq reads from HeLa cells [42] is shown as a control (gray). All intergenic regions in the human and mouse genomes were aligned relative to the annotated transcription start (TSS) or termination (TTS) sites of flanking genes. The robust average number of reads per 10 million uniquely mapped reads across all samples was then determined in 1 kb segments (RPKB) from the TSS or TTS, up to a distance of 30 kb, and the relative enrichment ratio in each segment was calculated by dividing by the median RPKB at distances more than 30 kb away from genes (baseline). Robust averages were calculated after removing the top 0.5% outliers, to avoid very highly expressed regions from having a disproportionate effect. (B) Same plots as in (A) for the combined reads from total RNA samples taken from human brain tissue and a universal human reference sample [35], uniquely mapped to the sense (blue) or antisense strand (yellow) relative to the neighboring gene region. (C) Histogram showing the distribution of correlation coefficients (red) between the read coverage in intergenic seqfrags and the nearest neighboring gene, across 11 human RNA-Seq samples. Read coverage was calculated as the number of reads per base per 10 million RNA-Seq reads across seqfrags and exonic regions of neighboring genes. Correlation coefficients were only calculated if the number of reads mapping to seqfrags and neighboring genes was greater than 10 in at least five out of eleven samples. The background distribution of correlation coefficients between seqfrags and randomly selected genes that met these thresholds is shown in comparison (gray). (D) Boxplot showing the correlation between the read coverage of intergenic transcripts and closest neighboring genes (red) or random genes (gray) across 11 human RNA-Seq tissue samples, as a function of their distance. (E) Representative example of intergenic transcription directly adjacent to the 3′ end of FAM114A1. The region with significant correlation is indicated by a red box. Mapped read coverage for the PolyA+ (black) and total RNA (blue) samples was standardized on a sequencing depth of 10 million reads and plotted in graphs scaled from 1- to 25-fold coverage.

We next sought to gain further insight into the nature of dark matter seqfrags, focusing mainly on intergenic regions to avoid possible interference from unprocessed RNAs in introns. Potential sources of seqfrags in intergenic regions include 5′ and 3′ extensions of known genes, aberrant termination products, pasRNAs, and novel genes. We therefore began our characterization of intergenic seqfrags by examining their relationship to neighboring genes. In both human and mouse PolyA+ RNA-Seq data, we observed that the average read density in intergenic regions is dramatically higher near the starts and ends of annotated genes (Figure 3A) and can extend up to a distance of ∼10 kb from both the transcription start and ends. We also observed bias towards genes in our tiling array analysis (unpublished data), as did a previous analysis using tiling arrays [8], but this study found the bias to be equal between 5′ and 3′ ends. In RNA-Seq data, the effect is stronger at the 3′ compared to the 5′ end of genes. Most transcripts at 3′ ends are consistent with alternative cleavage and polyadenylation (APA) site usage and unannotated UTR extensions of genes [16] or 3′ associated RNAs [12], rather than new exons, since in our splicing analysis (see below) we found very few instances of 3′ intergenic seqfrags linked to new 3′ exons (unpublished data). The increased number of transcripts at the 3′ end of genes is consistent with observations that RNA polymerase II can remain associated with DNA for up to 2 kb following the annotated ends of known mRNAs [40].

To determine the strand of origin of the positionally biased intergenic transcripts and to assess whether this bias was limited to PolyA+ RNA, we examined additional available sequencing-based transcriptome datasets. These included strand-specific RNA-Seq data from human rRNA-depleted whole brain and universal reference RNA [35], as well as from mouse brain PolyA+ [41] and rRNA-depleted total RNA (NCBI short read archive, SRX012528). We also incorporated data from CAGE-tag [12] and Paired-End diTag (GIS-PET) sequencing studies [42], which specifically targeted transcript ends. In all these datasets we find that most reads originate from known exons (Table S3), and among intergenic reads we find the same striking increase in read frequency in intergenic regions proximal to genes (Figure 3B, Figure S1, Table S3) as in PolyA+ samples. The enrichment of CAGE tags is consistent with peaks found at both the 5′ and 3′ ends of genes [12], and the majority of transcripts at the 3′ end of genes are in a sense orientation relative to the neighboring genes (Figure 3B). While CAGE tags are also enriched at 3′ ends of genes in the same orientation, the effect is less pronounced compared to RNA-Seq reads, suggesting that a significant number of transcripts in these regions result from alternative termination of protein-coding genes. Transcripts in intergenic regions flanking TSSs are approximately equally distributed between the sense and antisense strand (Figures 3B, S1A, and S1B), consistent with divergent transcription from promoter regions [12],[20]–[24], as well as unannotated 5′ transcript ends.

To examine the relationship between genes and gene-associated transcripts in greater detail, we next determined whether the increased sequence coverage of seqfrags in intergenic regions flanking genes correlated with the coverage of genic trancripts across the 11 human PolyA+ RNA-Seq samples (the same analysis could not be done for the mouse data, as the number of available samples was too low to reliably estimate correlations). To this end, we first identified intergenic seqfrags by merging overlapping RNA-Seq reads from all human samples and then determined the sequence coverage for seqfrags and genes in each sample. Figure 3C shows that the correlation in coverage between intergenic seqfrags and neighboring genes is much higher than it is for randomly selected genes, indicating that expression in intergenic regions is positively associated with that of the flanking genes. This effect is strongest up to a distance of 10 kb from the gene but persists to a lesser degree over larger distances (Figure 3D). After setting a threshold of *p*<0.05, based on how often the correlation coefficient between a given seqfrag and neighboring gene was expected to occur at random (Materials and Methods), we find a significantly increased correlation with intergenic seqfrags for 2,970 annotated genes, 934 of which remain after multiple testing correction (Table 1). Consistent with the increased read frequency at 3′ ends of genes, the number of genes with correlated intergenic seqfrags at the 3′ end is 3-fold greater than at 5′ ends of genes (Table 1). Many of the correlated seqfrags at 3′ ends are directly adjacent to the annotated genes (see Figure 3E for a representative example), adding further support to our hypothesis that many of these transcripts are linked in their expression. Additionally, we found a small number of extensions at larger distances, which are consistent with unannotated novel 3′ and 5′ exons (unpublished data and see below).

**Table 1:** Human transcripts with significantly correlated 3′ and 5′ seqfrags.

		Transcripts		Seqfrags	
Correlation Cutoff	Category	Counts	Fraction	Counts	Fraction
*p*≤0.05	All	2,970		6,109	
	3′ end	2,074	69.8%	4,612	75.5%
	5′ end	994	30.2%	1,497	24.5%
FDR ≤0.05	All	934		1,474	
	3′ end	698	74.7%	1,145	77.7%
	5′ end	251	26.9%	329	22.3%

The total number of genes with correlated 5′ and 3′ intergenic seqfrags is likely underestimated in our analysis, as a minimum number of sequence reads in each sample are needed to calculate a correlation coefficient. Many transcribed intergenic regions detected at very low coverage had to be excluded from the correlation analysis, even though these low coverage regions are clearly enriched in regions flanking known genes (Figure S2). Consequently, some positional bias is still observed after removing the regions identified in this analysis (unpublished data), and correlated transcription in regions flanking genes is likely far more widespread. This is particularly relevant because while the 10 kb flanking regions make up only ∼18% of the total intergenic area, they account for as much as 78% of the intergenic reads in human and mouse PolyA+ RNA. The same trend holds true for CAGE and GIS-PET datasets, as well as RNA-Seq datasets from rRNA-depleted human total RNA (Table S3). Although gene-flanking regions in rRNA-depleted mouse brain total RNA accounted for only 30.7% of intergenic reads, further inspection revealed that most of the reads outside these regions were linked to a small number of seqfrags (21) with excessive read counts (>10,000) confined to a small area (5 kb). This strongly suggests that there are a very small number of unannotated specific transcripts expressed at high levels, and after excluding these outliers, 71.1% of intergenic reads are found near genes (Table S3). The majority of intergenic dark matter transcripts are therefore linked to annotated protein-coding genes, either as extended transcripts or separate noncoding transcripts such as pasRNAs.

### Intergenic Regions Harbor a Limited Number of Novel Transcripts

**Figure 4:**
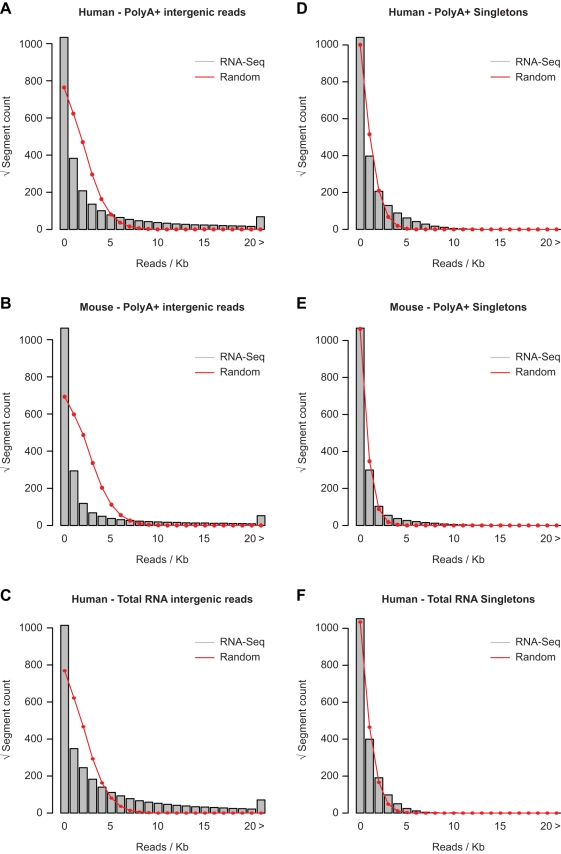
Evidence for specific expression in intergenic regions. Rootograms of the distribution of the number of the total number of RNA-Seq reads per kb of trimmed intergenic sequence for the combined (A) human PolyA+, (B) mouse PolyA+, and (C) human total RNA sequence data (gray bars), in comparison to the expected random distribution for the same number of reads (red lines). Ten kb intergenic regions flanking known gene annotations were excluded from the analysis. (D, E, and F) Same as (A), (B), and (C), but considering only intergenic transcribed regions with single-read coverage (singletons). The derived random distribution was adjusted accordingly.

Even when combining RNA-Seq data from all human or mouse tissues, read coverage in intergenic regions is very low (Figure 2B, 2C). To determine whether intergenic seqfrags are the result of low-level random background initiation, or whether they instead derive from a limited set of unannotated transcripts, we investigated the RNA-Seq read distribution in these regions. If the low-coverage intergenic seqfrags are indeed due to a uniform level of background initiation, reads should be spread evenly and the number of reads per kb of intergenic sequence should follow a random (Poisson) distribution. Given the observed transcriptional bias in regions flanking genes, we only considered intergenic regions that were at least 10 kb away from annotated genes (corresponding to ∼82% of all intergenic sequence). These trimmed regions account for 0.8% of the total number of reads in the human PolyA+ RNA-Seq data (1.64% for mouse), with an average coverage that is 9.4-fold lower than in intronic regions (3.3-fold for mouse). We find a clear departure from a random distribution in the trimmed intergenic regions of both species (Figure 4A, 4B), including several thousand loci with greater than 20 reads, which should not occur under our null hypothesis. We also independently assessed seqfrags that are supported by only a single RNA-Seq read in one tissue (“singletons”), which account for ∼70% of transcribed area in the trimmed intergenic regions in the human and mouse genomes. The distribution of singleton seqfrags is much closer to the random distribution (Figure 4D, 4E), although some deviation still persists for these low-coverage regions. To exclude that our observations are due to an inherent bias in cDNA library amplification or sequencing, e.g., due to GC content, we repeated the same analysis for an equal number of genomic DNA-Seq reads from HeLa cells [43] or a pool of human sperm DNA from four donors [44]. Both of these datasets were similarly generated on an Illumina genome analyzer and closely follow a random distribution (Figure S3). Taken together, these results indicate that while most reads >10 kb away from annotated genes are placed in a way that resembles random distribution across the genome, some have a non-random character, including several thousand regions with high read coverage that may be derived from unannotated novel transcripts.

To estimate the proportion of intergenic regions transcribed above background levels, we selected all 1 kb regions with a significantly higher read count compared to the random distribution (*p*<0.05) for all reads, or singleton reads only. At the lower thresholds based on singleton read frequencies, 3.0% (39.1 Mb) and 0.9% (11.4 Mb) of trimmed intergenic regions contain transcripts in the human and mouse genomes, respectively, decreasing to 1.2% (15.8 Mb) and 0.42% (5.25 Mb) at the more stringent thresholds. The increased area in the human compared to the mouse genome is consistent with the broader range of tissues assayed by RNA-Seq. The fraction of trimmed intergenic regions with significantly increased read counts is higher in human total RNA compared to PolyA+ RNA (Figure 4C, 4F): 4.1% (53.9 Mb) or 2.5% (32.8 Mb) at the lower and higher stringency levels, respectively. Considering that the total RNA sequence data was derived from a smaller sample set, this suggests that there are additional unprocessed and/or noncoding transcripts in intergenic regions not detected in PolyA+ RNA.

**Figure 5:**
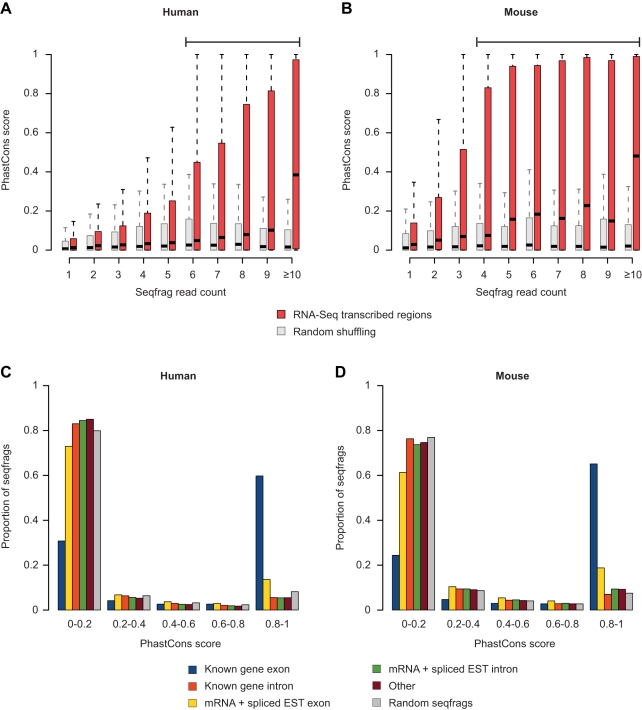
Seqfrags with read counts above background are conserved at the sequence level. Distribution of maximum PhastCons conservation score measured across seqfrags mapping to trimmed intergenic regions in the pooled (A) human and (B) mouse RNA-Seq samples as a function of read coverage (red). PhastCons scores were obtained from the UCSC genome browser and reflect the degree of conservation in multiple alignments of the human and mouse genomes with 18 and 20 other mammalian species, respectively. Conservation scores obtained from a random shuffling of seqfrag positions within trimmed intergenic regions are shown in gray for comparison. (C and D) Bar plots indicating the PhastCons score distribution for seqfrags mapping to different genomic regions. The bars are color-coded according to the class of seqfrags (legend), with the score distribution for randomly mapped seqfrags shown in gray.

We also applied an additional threshold to identify putative novel exonic regions in the trimmed intergenic areas, selecting for seqfrags with a PolyA+ RNA-Seq read count greater than or equal to that of the top 5% of seqfrags detected in known introns (6 reads for human and 4 for mouse). At these thresholds we find 16,268 potentially “exonic” seqfrags in human (spanning 2.5 Mb) and 11,533 in mouse (spanning 0.66 Mb), which account for 56.9% and 87.4% of the reads in the trimmed intergenic regions in each organism, respectively. The area covered by the putative exonic seqfrags is 3.8% of the total area covered by seqfrags overlapping known exons in the human genome and 1.4% for the mouse genome. The putative exonic seqfrags tend to be well conserved at the sequence level compared to a random selection of intergenic sequences (Figure 5A, 5B), as judged by PhastCons conservation score based on multiple alignments among 18–22 mammalian genomes. This is significant, considering that the overall conservation for intergenic and intronic reads is close to random (Figure 5C, 5D). Taken together, our results show that a limited number of conserved novel exonic seqfrags can explain the majority of intergenic transcript mass detected in PolyA+ RNA, with a small proportion of low-level transcripts over a broad area that may be due to random initiation events.

### Global Splice Junction Analysis Identifies New Transcript Structures

We next attempted to identify novel transcript structures by detecting splice junctions between transcribed regions in the genome using Tophat [45]. Tophat uses a two-stage approach that first aligns unspliced RNA-Seq reads to the genome to identify transcribed areas, which are then examined in the second stage to identify junction sequences spanning all possible 5′ and 3′ combinations of these regions, using the reads that could not be mapped in the first stage. The main advantage of this approach is that it does not require a predefined set of annotated exons and it can therefore identify splicing between unannotated regions of the genome. Moreover, as the analysis takes the canonical splice junction donor and acceptor sites (GT-AG) into account, it is possible to determine the strand of origin for each junction, despite the fact that the PolyA+ RNA-Seq data used in this study were not generated in a strand-specific manner. We restricted our analysis to human samples, since we found the reads in the mouse dataset to be too short to reliably detect junction sequences.

Overall, we found 160,516 unique splice junctions in the 11 PolyA+ human RNA-Seq samples, 151,708 (94.5%) of which can be classified as “known,” meaning that they span any two exons within a single annotated transcript (Table S4). The remaining 8,808 novel junctions involved a single known exon or spanned two unannotated regions in the genome. In total, we could detect 57.8% of all exons in the combined set of gene annotations by at least one junction. Only 300 junctions bridged exons between transcripts, and almost all mapped to tandem-repeated regions in the genome (Table S5). Considering the high degree of sequence similarity between the repeated regions, some of these are presumably due to mapping inaccuracies. A significant proportion of bridging junctions (47%, 25% with confirmed deletions) also overlap regions with validated copy number variations (CNVs) that are common in the general population [46], suggesting that others may result from gene fusions following deletion events. These findings further argue against pervasive transcription to the extent reported in previous studies.

We assessed the false positive rate in the detected junctions by randomizing the sequences of potential splice junction reads and determined it to be 0.054% for paired-end reads and 2.7% for single-end reads (see Materials and Methods). The higher accuracy for paired-end reads demonstrates the considerable advantage of using longer reads to accurately assess splice junctions. Indeed, we found that the shorter 32 mer reads are particularly sensitive to false positive detections due to the presence of low-complexity regions and PolyA/T repeats, and we therefore applied additional filtering steps to exclude the affected junctions (see Materials and Methods for details). The longer read lengths of the paired-end compared to single-end RNA-Seq samples, combined with a 4-fold increase in sequencing depth, also resulted in a more than 3-fold higher splice junction detection rate.

The fact that short RNA-Seq reads typically cover only a single junction between exons makes it difficult to determine which combinations of alternative splice junctions correspond to transcripts observed in vivo. We therefore instead focused on identifying transcriptional units (TUs) that represent the aggregate assembly of all connected splice junctions. Thus, a completely reconstructed TU for an annotated gene will comprise the full complement of exonic regions, though these may be used in different configurations in alternatively spliced transcripts. Splice junctions were considered connected if they were directly adjacent to each other on the same strand, arranged in a head-to-tail configuration, and (i) the “facing” junction ends overlapped, or (ii) the complete region between facing splice junctions was transcribed, or (iii) facing junctions were within a distance of 200 bp (i.e., the approximate average exon size).

The vast majority of TUs we identified (91.2%) overlap with at least one exon of an annotated gene (Table 2), and 92.1% of exons in these TUs overlap known gene annotations (Table 3). We also detected 3,451 unannotated internal exons in 2,720 genes, as well as 723 and 370 unannotated 5′ and 3′ exons, affecting 544 and 290 genes, respectively. Among the TUs that are not connected to known gene annotations (i.e., independent TUs), 1,259 map to intergenic regions, the majority of which (82.6%) consist of a single junction. Only a minor fraction of independent TUs (4.8% of the total number of TUs) overlap genic regions on the sense or antisense strand. As it is possible that additional rare splice junctions are not detected in our analysis, some independent TUs overlapping genes in the sense direction may yet turn out to be connected to the gene they overlap. The majority of novel exons in the reconstructed TUs overlap with exons from the UCSC mRNA and spliced EST tracks (Table 3), providing further evidence that they are derived from true splicing events. A small number (73) further overlap exons predicted by Wang et al. [16], which were derived from an analysis of splice junctions associated with computationally predicted exons. Taken together, our findings confirm that the vast majority of spliced transcripts in PolyA+ RNA are linked to known gene annotations and argue against widespread interleaved transcription of protein-coding genes in the human genome. The full set of TUs and junctions has been made available on our supplementary website (http://hugheslab.ccbr.utoronto.ca/supplementary-data/hm_transcriptome/).

**Table 2:** Overview of transcript units identified in human RNA-Seq samples.

	Transcript Units		Breakdown by # of Exons		
Category	Count	Fraction	Exons	Transcript Count	Fraction
Exon overlap with known gene	29,029	91.2%	2	9,546	32.9%
			3	4,414	15.2%
			>3	15,069	51.9%
Non-exon gene overlap, sense strand	475	1.5%	2	422	88.8%
			3	29	6.1%
			>3	24	5.1%
Non-exon gene overlap, antisense strand	1,055	3.3%	2	927	87.9%
			3	94	8.9%
			>3	34	3.2%
Intergenic	1,259	4.1%	2	1,040	82.6%
			3	168	13.3%
			>3	51	4.1%

**Table 3:** Exon overview for transcript units in human RNA-Seq samples.

	Exons		EST + mRNA Overlap	
Category	Count	Fraction	Count	Fraction
Known gene	174,693	94.2%		
New exon for known gene	4,544	2.5%	3,060	67.3%
Internal	3,451	1.9%	2,291	66.3%
External, 5′ end	723	0.4%	523	72.3%
External, 3′ end	370	0.2%	246	66.4%
Overlapping known gene	3,364	1.8%	2,223	66.0%
Sense strand	1,069	0.6%	609	57.0%
Antisense strand	2,295	1.2%	1,614	70.3%
Intergenic	2,821	1.5%	1,686	60.0%

### Characterization of Novel Exons and Multi-Exon Transcript Units

**Figure 6:**
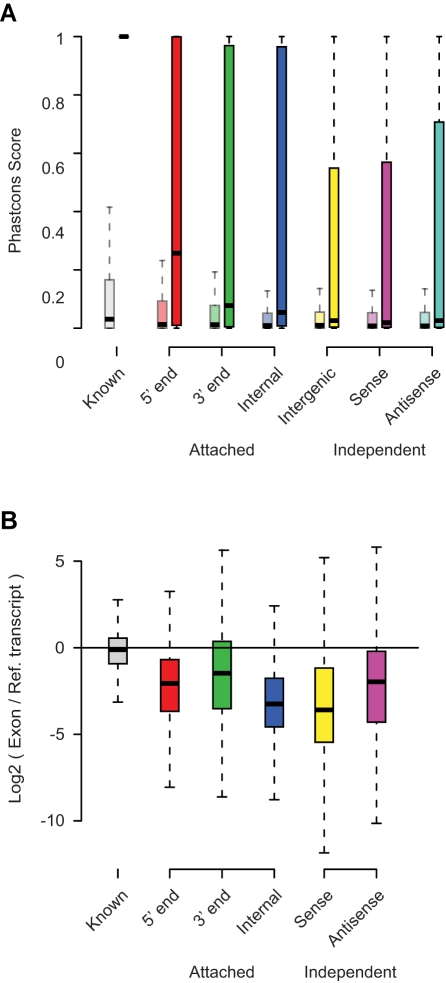
Conservation and usage of human TU exons. (A) The distribution of PhastCons scores for novel exons in each category as in (A) (darker bars), compared to the distribution of scores from the same set of exons after random reshuffling their positions in the genome (lighter bars). (B) Plot of the ratios between the read coverage of novel exons (calculated in RPB) and the genes they are associated with, either by overlap (sense or antisense) or as additions to known gene structures (5′ end, 3′ end, and internal). The ratios for predicted exons overlapping exons of known gene structures are shown in comparison.

To further characterize the 4,544 novel exons connected to existing transcripts, as well as the 2,789 novel independent TUs (i.e., multi-exon transcripts), we assessed their expression levels, degree of conservation, and coding potential. As expected, novel exons detected as part of TUs that overlap annotated transcripts show evidence of increased conservation compared to randomly positioned exons (Figure 6A). Consistent with our analysis, a significant proportion of these exons overlap with Exoniphy predictions of evolutionary conserved protein-coding exons [47], most notably for novel 3′ (20.5%) and 5′ exons (18.9%) (Table S6A). The degree of overlap was significantly higher compared to random selections from intergenic regions (*p*<0.0001). In contrast, we observed little overlap with conserved RNA secondary structures as predicted by the Evofold [48] and RNAz algorithms [49] (Table S6A). We further examined whether the novel 5′ exons overlapped regions of open chromatin that typically mark regulatory regions [50]–[52] and which can be identified using digital DNase I hypersensitivity assays [53]. To this end, we used publicly available genome-wide data on DNase I hypersensitivity hotspots generated by the UW ENCODE group for 11 cell lines [54]. Consistent with their expected association with promoter regions, we found that the majority of novel 5′ exons overlapped the complete set of DNase I hypersensitivity zones identified by the HotSpot algorithm [53] in all 11 cell lines, as well as a more restricted set that only included hotspots found in both replicates for 8 cell lines (*p*<0.0001) (Table S6A).

**Figure 7:**
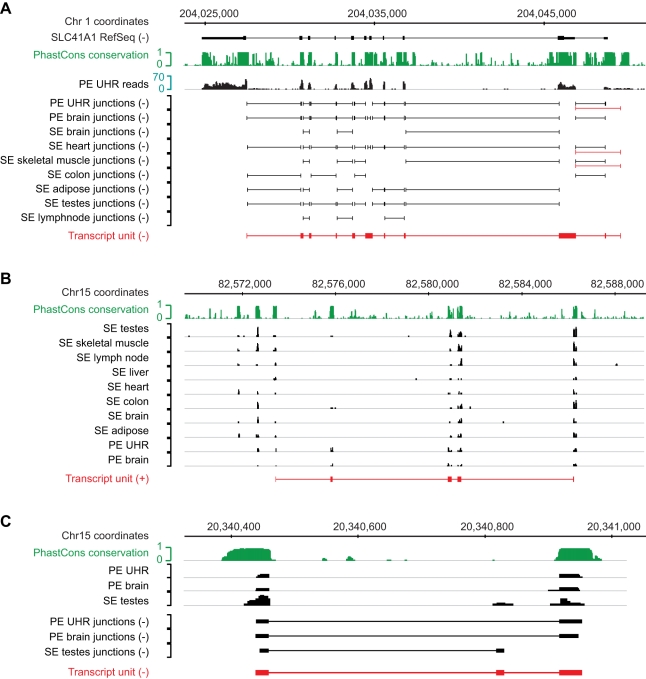
Examples of identified TUs. (A) Evidence for the presence of an alternative promoter at the human SLC41A1 gene. Splice junctions connecting to the alternative promoter region are indicated in red. Mapped RNA-Seq data for the UHR paired-end (PE) read sample is shown for reference (black). The PhastCons conservation track scores were based on multiple alignments of 28 vertebrates. (B) Protein-coding TU detected in an intergenic region on chromosome 17, with high similarity to the elongation factor Tu GTP binding domain. The two additional upstream transcribed regions may be part of the same transcript, though no junction sequences were detected. (C) Intergenic TU (red) detected on chromosome 15 based on junctions in the PE brain, PE UHR, and SE testes RNA-Seq samples.

Most of the novel exons are expressed at lower levels compared to the other exons of the gene they are linked to, which suggests that they derive from low-frequency alternative splicing events in the tissues we examined (Figure 6B). Indeed, we find direct evidence of alternative splicing for 2,526 (73%) of the novel internal exons and 2,370 of these (94%) are overlapped by junctions that bypass the novel exon. For novel exons at the 5′ and 3′ termini there is direct evidence for alternative splicing for 310 (43%) and 144 (39%), respectively. Among these are 145 cases of clear alternative promoter usage, where we find splice junctions between internal exons and the annotated promoter, as well as alternative junctions that link to a more distal promoter (Table S7). Figure 7A shows an example of one such alternative promoter for the SLC41A1 gene, encoding a solute carrier family protein.

In contrast to many of the transcribed fragments reported in tiling array studies, we find evidence for higher overall conservation for exons in independent TUs in intergenic regions, and those overlapping genes on the sense or antisense strand (Figure 6A). We assessed the coding potential of the independent TUs using a support vector machine classifier that incorporates quality measures of the available open reading frames (ORF) and blastx results [55]. Larger independent TUs with three or more exons show a general tendency to be coding: 60.8% in the case of intergenic TUs, and 70.8% and 41% for TUs overlapping genes on the sense and antisense strand, respectively. An example of a coding transcript with a translated ORF that has high sequence similarity to the elongation factor TU GTP binding domain is shown in Figure 7B. Some of the other translated TUs with clear similarities to existing proteins have stop codon mutations within the ORF, indicating that they could be pseudogenes.

None of the smaller intergenic TUs (containing only a single splice junction) were classified as coding. We note, however, that it is challenging to reliably detect the coding potential of small transcript fragments, and some of the TU fragments may in fact be part of larger coding transcripts. Indeed, when we extended the independent TUs by incorporating seqfrags overlapping the flanking junction sequences in the detected TUs, the proportion of potential coding transcripts increased to 8.3% for TUs overlapping gene regions on the antisense strand and to ∼17% for TUs overlapping genic regions on the sense strand and intergenic TUs. Moreover, we find a significant overlap with Exoniphy predictions of coding exons, ranging between 10.5% for intergenic TUs and 21% for antisense TUs (Table S6B). Further investigation will be required to characterize these smaller TUs.

Even among the larger intergenic TUs with three or more exons, there is a subset of 116 transcripts that appear to be noncoding and are thus potential human lincRNAs, one example of which is shown in Figure 7C. The fact that we could not perform a comprehensive splice junction analysis in the mouse RNA-Seq data precludes us from making a detailed comparison with the previously identified mouse lincRNAs [19], however we do find a significant overlap between 95 of the mouse intergenic seqfrags with a read count above background and 30 of the lincRNA regions (Table S8A,B). The observation that there is little overlap (0%–1%) between reconstructed TUs and Evofold and RNAz predictions (Table S6B) suggests that most transcripts identified here do not fold into conserved RNA structures. In summary, our results reveal novel alternatively spliced exons and promoters in the human genome that are used at relatively low frequencies, as well as new lincRNA candidates.

### Many Transcripts in Intergenic Regions Distal from Genes Are Short, Unspliced, and Associated with DNase I-Hypersensitive Regions

**Figure 8:**
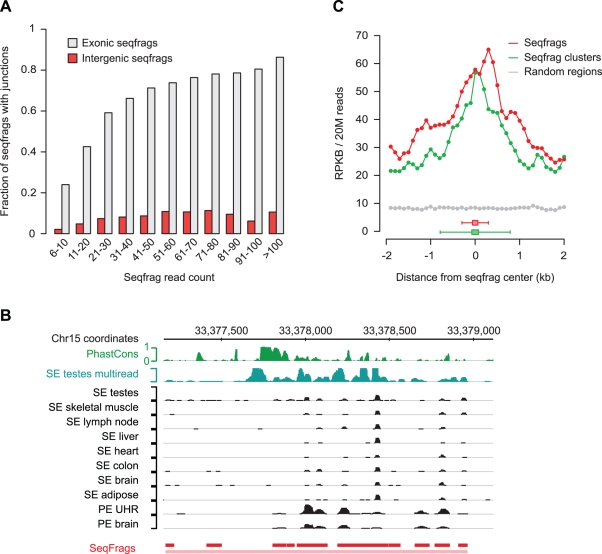
Most intergenic transcripts are unspliced and associated with open chromatin. (A) Relationship between read count and the fraction of seqfrags with at least one identified junction sequence for seqfrags in exonic (gray) or trimmed intergenic (red) regions. (B) Cluster of ubiquitously expressed seqfrags derived from uniquely mapped reads on chromosome 15. An additional track with multireads from SE testes RNA-Seq data (blue) shows that many of the uniquely mapped seqfrags are part of a larger, continuously transcribed region. (C) Digital DNase I hypersensitivity profiles in RA-differentiated SK-N-SH cells for 11,416 seqfrags (red) and 5,819 seqfrag clusters (green) expressed in human brain. Hypersensitivity is shown as the average density of in vivo cleavage fragment reads per kb (RPKB, normalized to 20 million reads) across all seqfrags or clusters, measured in 100 bp windows flanking the center position of each seqfrag or cluster up to a distance of 2 kb. The DNase I hypersensitivity at random positions in intergenic regions is shown as a control (gray). The box-and-whisker plots at the bottom of the graph indicate the median (box) and the 95th percentile (whiskers) of the seqfrag- (red) and seqfrag cluster size range (green).

Only a small proportion (3.6%) of the 16,268 human intergenic seqfrags we identified with a read count above background were found to be part of TUs, which was surprising given that we could identify splice junctions for the majority of seqfrags in annotated exons. The lack of junctions connecting intergenic seqfrags cannot simply be explained by a reduced detection rate due to lower read counts compared to exonic seqfrags, as the proportion of intergenic seqfrags with detected junctions is consistently lower even at high coverage levels (Figure 8A). We therefore conclude that the majority of intergenic seqfrags are derived from unspliced single-exon transcripts. However, the remaining 15,646 human seqfrags that are not part of TUs are often spaced closely together, suggesting that they may be part of a single transcript, or are processed individually from larger precursor transcripts. Indeed, in many cases the intervening sequence between consecutive seqfrags is classified as transcribed when allowing reads mapping to multiple positions in the genome (see, for example, Figure 8B). When we group neighboring seqfrags with a maximum gap of 500 bp, 8,536 seqfrag clusters remain in human (7,976 of which show no evidence of splicing) and 5,506 in mouse.

We used the support vector machine classifier and Exoniphy predictions of coding exons, described above, to examine the coding potential of the unspliced intergenic seqfrags. Only 1.4% and 3.5% of human and mouse intergenic seqfrags with a read count above background overlap Exoniphy predictions, respectively (Table S8A,B). Moreover, out of the top 5% largest human intergenic seqfrags, ranging in size between 0.4 and 3.8 kb, only 12% were classified as coding. Taken together, these observations strongly suggest that the majority of the small intergenic seqfrags we identified are noncoding. As in the case of intergenic TUs, these transcripts also display little overlap with Evofold and RNAz regions.

The most striking property of the unspliced seqfrags is their strong association with open chromatin: 6,407 out of the 15,646 (40.9%) human intergenic seqfrags overlap with DNase I hypersensitivity hotspots identified in one of the 11 cell lines that were assayed, 3.4-fold more than would be expected by chance (Table S8A). Figure 8C shows a clear enrichment in tags from hypersensitive sites for RA-differentiated SK-N-SH neuroblastoma cells across the full length of brain-expressed seqfrags. Moreover, the typical size of the unspliced seqfrags (median 111 bp) is smaller than that of the DNase I-hypersensitive regions (median 248 bp), and unlike coding transcripts and other ncRNAs, many of the seqfrags appear to be contained entirely within the DNase I-hypersensitive regions. We expect that the true number of seqfrags associated with DNase I hypersensitive regions may be larger, considering that the cell lines assayed only account for a small selection of the cell types represented in the tissues and cell types assayed by RNA-Seq. Thus, these analyses reveal the existence of thousands of small intergenic transcripts associated with open chromatin.

## Discussion

In contrast to earlier studies based on oligonucleotide tiling array analysis of RNA [1]–[9], GIS-PET [9], and RACE-tiling arrays [9], but consistent with other RNA-Seq studies [16],[33],[36]–[38], we find that the proportion of dark matter transcripts among polyadenylated RNA from a large variety of different tissue types is small. Our comparison between tiling arrays and RNA-Seq data from the same tissues indicates that tiling arrays are ill-suited to accurately detect transcripts expressed at low levels. The major fraction of nonexonic transcripts in RNA-Seq data is associated with known genes and includes thousands of new alternative exons and hundreds of alternative promoters. However, we do not find evidence for widespread interleaved transcripts as previously described [9]; virtually all exon-exon junctions detected correspond to junctions within the same gene. Aside from new exons, most of the transcripts that are within or proximal to known genes can be explained as pasRNAs or terminator-associated RNAs, pre-mRNA fragments, or by alternative cleavage and polyadenylation site usage. The relatively small fraction of seqfrags that are not associated with known genes corresponds strongly to DNase I-hypersensitive regions. Altogether, we propose that most of the dark matter transcriptome may result from the process of transcribing known genes. Pervasive transcription of intergenic regions as described in previous studies occurs at a significantly reduced level and is of a random character.

The intergenic regions that are transcribed above background consist of a mix of both coding and noncoding transcripts. In contrast to the extensive intergenic transcription reported in tiling array studies, we found relatively few transcripts in these regions (16,268 seqfrags expressed above background levels in human and 11,533 in mouse). These numbers may be smaller, as some adjacent seqfrags may be parts of a single transcript that contain regions with sequence mapping ambiguities, or they may be larger as more tissues and cell types are surveyed.

The fact that non-exonic transcripts do not overlap with Evofold or RNAz regions argues against widespread roles as structural RNA. The most compelling support that these transcripts may have an independent function comes from the fact that they overlap with DNase I hypersensitive regions and that, unlike the many transcripts found by tiling array studies and from deep sequencing of subtracted cDNA libraries [11], the transcripts found by RNA-Seq show a significantly higher degree of conservation between species. We note, however, that these same two properties are consistent with low-level transcription from enhancers. Indeed, in yeast, it is known that placement of a strong activating transcription factor binding site in random regions of the genome results in the formation of a promoter [56]. Thus, single-exon intergenic seqfrags may represent the analog of pasRNAs for enhancers.

Our findings are based primarily on analysis of PolyA+ enriched RNA; however, our conclusions are corroborated by CAGE tags, GIS-PET, and RNA-Seq analysis of rRNA-depleted total RNA. Similar conclusions to ours were also reached in an independent RNA-Seq analysis of rRNA-depleted human total RNA (G. Schroth, pers. communication). It does not appear as if additional sequencing would substantially alter our conclusions, since coverage bias towards known exons increases with the number of reads. Moreover, while RNA-Seq analysis of PolyA+ RNA biases against very long and very short RNAs, this would not be expected to affect our ability to detect the widespread and pervasive transcription reported previously. Nonetheless, analysis of further tissues and cell types would be expected to identify additional intergenic ncRNA seqfrags that are more abundant but expressed in rare or specialized cell types. It is also likely that total RNA harbors additional transcripts not seen in PolyA+ enriched RNA and that are not evident in current total RNA-Seq analyses due to limitations in read counts.

A major remaining question is the possible function of the novel intergenic transcripts, if any. Undoubtedly, there are many functional ncRNAs remaining to be characterized [57]. However, we and others have emphasized that expression, conservation, and even localization and physical interactions of these RNAs do not constitute direct evidence for function [32]. Promoters and terminators are known to produce transcripts that appear to be associated primarily with the mechanics of gene expression and do not have known independent functions. To be conservative, a null hypothesis should perhaps be that novel transcripts—particularly those that are small and low-abundance—are a by-product rather than an independent functional unit [58]. Searching for phenotypes caused by genetic perturbation may be the most useful approach to disproving the null hypothesis.

## Materials and Methods

### Sample Sources

Total and PolyA+ samples for tiling array hybridizations from pooled human and mouse heart, liver, testis, and whole brain tissues were obtained from Clontech (Table S9). All human RNA samples were derived from tissues of individuals that suffered sudden death. The human whole brain PolyA+ RNA used for paired-end sequencing came from a Microarray Quality Control (MAQC) sample (Ambion) that consisted of a mixture of RNA from 23 Caucasian males. The PolyA+ selected universal human reference sample (Stratagene) consisted of pooled RNA from 10 human cell lines (Adenocarcinoma, mammary gland; Hepatoblastoma, liver; Adenocarcinoma, cervix; Embryonal carcinoma, testis; Glioblastoma, brain; Melanoma; Liposarcoma; Histiocytic Lymphoma, hystocyte; Lymphoblastic leukemia, T lymphoblast; Plasmacytoma, B lymphocyte).

### Microarray Hybridizations

All RNA samples were DNase treated with 10 units of DNase I (Fermentas) per 50 ug of RNA prior to cDNA synthesis and purified with RNeasy spin columns (Qiagen) using a modified protocol that retains small RNAs <200 nt. Double stranded cDNA synthesis was done as previously described in Kapranov et al. [5]. Briefly, 9 ug of total RNA was reverse transcribed in a reaction that contained 1,800 units of SuperScript II enzyme (Invitrogen) and 83.3 ng of random hexamers and Oligo(dT) primers per ug of RNA. The cDNA was then used for second strand synthesis, after which the double-stranded cDNA (ds-cDNA) was purified using PCR purification columns (Qiagen) in combination with the nucleotide cleanup kit protocols. Following fragmentation and biotin labeling, 7 ug of ds-dDNA was hybridized per array.

### Mapping of Genomic Coordinates for Tiling Array Probes

The Affymetrix Human and mouse tiling arrays version 2.0R were originally designed for the NCBI genome assemblies v34 and v33, respectively, and were remapped to more recent genome builds (v36 for human and v37 for mouse) using BLAT [59], not allowing for any mismatches in the alignments. A small number of probes mapping to multiple locations in the genome were assigned a position that would conserve probe order relative to the original array design. In cases where this was not possible, the position on the same chromosome nearest to the original probe location was selected, or a match was randomly selected if none could be found on the same chromosome. In total, 99.5% of probe sequences could be remapped to the new mouse genome assembly, and for the human arrays this number was close to 100%. Updated bpmap files are available on request.

### Microarray Data Analysis

Arrays were scanned using an Affymetrix GeneChip scanner 3000 and raw probe intensities were obtained using the Affymetrix GeneChip Operating Software. Each array was quantile normalized against a reference genomic DNA hybridization using the Affymetrix Tiling Array Software v1.1 to obtain intensities corrected for probe sequence bias (Figure S4). The probe intensity data were further smoothed by calculating the pseudomedian of genomic DNA-normalized intensity values of probes that lie within a genomic sliding window around each probe [5]. The size of the sliding window was determined by the bandwidth parameter (BW) as follows: (2× BW) +1. Transcribed regions (transfrags) in tiling array data were selected as previously described [5], by joining positive probes together using three parameters: (i) an intensity threshold to select positive probes, (ii) the maximal distance (MAXGAP) that two neighboring positive probes can be separated by, and (iii) the minimal transfrag length (MINRUN). A range of BW, MAXGAP, and MINRUN parameter combinations were applied and used to assess precision and recall of exons in known transcripts.

### Illumina Sequencing Datasets

Libraries for paired-end sequencing were prepared according to the manufacturer protocols. After selecting for cDNA fragments with a size distribution around 200 bp, 50 bp on both ends were sequenced in an Illumina Genome analyzer II. Single-end RNA-Seq data with a read length of 32 nt for PolyA+ RNA for 8 human tissues from individual donors (Adipose, Brain (2×), Colon, Heart, Liver, Lymph Node, Skeletal Muscle and Testis) were obtained from a previous study by Wang et al. [16]. Twenty-five mer single-end read data for PolyA+ RNA from three mouse tissues (Brain, Liver, Skeletal muscle) were taken from Mortazavi et al. [36]. Both literature datasets were produced following similar protocols that included a fragmentation step followed by a size selection for fragments of ∼200 bp and sequencing on an Illumina Genome analyzer. All paired-end RNA-Seq data are available on our supplementary website (http://hugheslab.ccbr.utoronto.ca/supplementary-data/hm_transcriptome/).

### Mapping of Unspliced RNA-Seq Reads to Reference Genomes

Single-end read RNA-Seq data were mapped to the NCBI human and mouse genome assemblies v36 and v37, respectively, using Seqmap v1.0.10 [60]. Several parameter settings were tested, and the maximum number of uniquely mapped reads (best unique hit) was obtained by restricting the read length to the first 25 bases and allowing for only one mismatch (Figure S5). These settings were subsequently used for all single-end read mappings. Paired-end reads from human brain and UHR samples were split and independently mapped using bowtie [61], selecting only the unique best hits from alignments that had a maximum of two mismatches in the seed sequence (first 28 bases) and an overall sum of mismatch phred quality scores no greater than 70. Single-end reads or tags from strand-specific datasets (Table S3, Figure S1) were also mapped using bowtie [61] to maintain strand information.

For the overlap analysis with known gene annotations, we combined the following tracks from the University of California Santa Cruz (UCSC) genome browser: UCSC known genes, Refseq genes, ENSEMBL genes, RNA genes, miRNAs, and snoRNAs (February 2009). In addition, mRNA and spliced EST tracks were obtained from the same source (September 2009) for a secondary mapping of seqfrags or sequence reads that did not match known gene annotations. Non-redundant sets of genes, mRNAs, and spliced ESTs were prepared by merging overlapping features, where the resulting exonic regions were defined as the union of exons in the source annotations and introns as the intervening regions between merged exons. For the calculation of the proportion of reads accounted for by each annotation category, reads were considered exonic if they partially or fully overlapped a merged exon, and intronic or intergenic if they were fully contained in these respective regions. The proportion of transcribed area was calculated by intersecting the genomic coordinates of continuously transcribed genomic regions (seqfrags) and the various genome annotation categories.

### Conservation of RNA-Seq Regions

PhastCons [62] conservation scores for the human and mouse genomes were obtained from the UCSC website and were based on multi-species alignments of 18 (hg18-phastCons18way) and 20 (mm9-phastCons20way) placental mammals, respectively. Conservation scores were assigned to each seqfrag by taking the maximum PhastCons score in the genomic region covered by the seqfrag. For comparison purposes, a background score was determined for each seqfrag in the same manner, after reassigning seqfrags to random positions in the genome or within intergenic regions.

### Correlation Analysis for Positional Bias

To calculate Pearson correlation coefficients between the expression levels of transcribed intergenic regions and the closest neighboring genes, overlapping mapped reads from all 11 human RNA-Seq samples were first merged into seqfrags. For each seqfrag, the nearest neighboring known transcript with read coverage in at least five RNA-Seq samples was then selected from the full set of transcripts in the UCSC known gene, Refseq gene, ENSEMBL gene, RNA gene, miRNA, and snoRNA tracks. In case multiple transcripts were found at the same distance (e.g., alternatively spliced transcripts), the transcript was selected that maximized the number of available data points for correlation analysis. The transcript expression levels in each tissue were defined as the median read coverage per kb of exon sequence and further adjusted for the difference in sequence coverage between RNA-Seq samples. Read coverage for intergenic seqfrags was determined analogously. Pearson correlation coefficients between transcript and seqfrag expression levels were only calculated if the read coverage for both the seqfrag and transcript were above zero in at least five of eleven samples, and all other intergenic seqfrags were removed from the analysis. The significance of the correlations was determined by comparing seqfrag expression levels to those of 1,000 randomly selected genes that met the same cutoff criteria. Nominal *p* values were defined as the proportion of random permutations where the correlation coefficient exceeded the observed correlation with the closest neighboring gene. Nominal *p* values were further adjusted for multiple testing by applying a Benjamini-Hochberg FDR correction [63] using the multtest R package from Bioconductor [64].

### Assessment of the Intergenic Read Distribution

To determine whether RNA-Seq reads that map outside genes follow a random (Poisson) distribution, intergenic regions were divided into 1 kb segments and the total number of reads and the number of singleton reads in each segment was counted. Regions flanking genes up to a distance of 10 kb were excluded, as reads in these regions are more frequent, and correlated with known genes. For comparison, a random distribution was derived by sampling an equal number of uniquely mapped random reads with the same size distribution as the mapped RNA-Seq reads. To avoid a potential bias from the paired-end reads, we only mapped one of the reads in a pair. Comparisons between random and observed distributions were visualized in rootograms, which plot the square root of the number of segments as a function of the number of reads in each segment, allowing for a better assessment of differences at the tail of the frequency distribution.

### Splice Junction Discovery

Analysis of novel splice junctions was performed using Tophat [45], which uses a detection method outlined in Figure S6A. Briefly, Tophat searches for splice junctions by first mapping RNA-Seq reads to the genome to identify “islands” of expression, which are equivalent to seqfrags. In contrast to the mapping of unspliced RNA-Seq reads described above, Tophat allows multiple genomic matches for each read (up to a maximum of 40 copies) during this mapping step. Each expression island is then considered a potential exon and used to build a set of potential splice junctions, taking into account the canonical splice donor and acceptor sites (GT-AG) within each island and a small flanking region of 45 bp. Subsequently, each possible pairing of neighboring junction sequences up to a specified distance (determined by the maximum allowed intron size) is compared to the set of “missing” RNA-Seq reads that could not be matched to the genome in the first mapping step to identify sequences that span junctions. Islands with high coverage are also examined for internal junctions, to account for the possibility that the intervening intronic region between two highly expressed exons is fully transcribed at lower coverage. Paired-end reads were analyzed with Tophat version 1.0.10, which features improvements in splice junction detection specific to paired sequencing data by taking the distance between read pairs into account. In contrast, single-end read data were analyzed using Tophat version 0.8.3, as we found that this version offered greatly improved sensitivity for shorter unpaired reads.

Splice junctions in paired-end read data were mapped allowing for a maximum intron size of 500 kb, which is sufficient to encompass 99.99% of all introns and 99% of all transcripts in the complete set of annotated transcripts described above. The minimum required read match size at each junction end (i.e., anchor size) was set to 8 nt. Finally, the minimum isoform fraction was set to 0.15 to suppress junctions that were supported by too few alignments relative to the junction exons. The isoform fraction was calculated as S/D, where S is the number of reads supporting each junction and D is the average coverage of the junction exon with the highest coverage [45]. Splice junctions in single-end read data were mapped using the full 32 mer read length, rather than the shorter 25 mer reads used for the mapping of unspliced reads. The Tophat parameter settings for single-end reads were the same as for the paired-end reads, with the exception that the minimum anchor size was set to 11 and the intron size was set to 20 kb (sufficient to bridge the length of 93.98% of all annotated introns and 53% of all transcripts). The adjusted parameters for single-end read data increased precision due to the shorter read lengths (Figure S7), at the expense of a somewhat reduced ability to detect long-range splice junctions. Finally, junctions with identical sequence that mapped to more than one genomic location in both the single- or paired-end RNA-Seq data were dropped from the analysis. Alternative splicing events were defined as junctions that shared the same start position with another junction but ended at a different position, or vice versa.

### Estimation of False Positive Rate in Novel Splice Junctions

In order to estimate the proportion of false positives in the splice junction prediction, we adjusted Tophat to use a modified set of reads in the splice junction detection step. The initial read mapping stage to identify islands of expression was unchanged, but the sequence for the set of “missing” reads used in the second stage to detect splice junctions between islands was reversed (Figure S6A), resulting in a scrambled set of potential junction sequences with very similar sequence properties, in particular for low-complexity and repetitive regions. In addition, the pairing of reads in the paired-end dataset was randomized. With the modified sets of “missing” reads, 62 junctions were detected in the brain and 60 in UHR sample, corresponding to an estimated false positive rate of 0.054% for paired-end read samples at the selected analysis thresholds.

At 7.3%, false positive rates for single-end reads were significantly higher, consistent with the shorter read lengths. Further examination of junction sequences revealed an over-representation of PolyA and PolyT repeats in junction sequences of single- compared to paired-end read samples (Figure S8). We believe that enrichment of these repeats is due to a bias in mapping short reads sequenced from PolyA tails, and additional filtering steps were therefore applied to exclude junctions with a PolyA/T repeat size larger than 5. Moreover, any junction found to contain more than 20% low-complexity regions as assessed by the DUST algorithm (http://compbio.dfci.harvard.edu/tgi/software/) and repeatmasker (http://www.repeatmasker.org) was discarded. After applying these filters to the real and randomized junction set, the false positive rate for detection of splice junctions in the single-end read set was reduced to 2.7%.

### Assembly of Splice Junctions into Transcript Units

TUs were defined as described in the main text. Facing splice junctions arranged in a head-to-tail fashion were first assembled into tissue-specific TUs if (i) splice junction ends overlapped (ii) the complete region between facing splice junctions was transcribed or (iii) if facing splice junctions were within a distance of 200 bp (same range as the average exon size) (Figure S6B). TUs were then combined across tissues where TUs with at least one overlapping exon were merged to create a non-redundant set. Exons were detected either partially, with junctions on only one side (e.g., 5′ and 3′ terminal exons), or completely, with supporting junctions defining boundaries on both sides.

### Assessment of Coding Potential of Novel TUs

The coding potential of novel transcript fragments was assessed using a support vector machine classifier [55] that assesses the protein-coding potential based on several sequence features that incorporates quality assessments of the predicted ORF as well as BLASTX comparisons with the NCBI non-redundant protein database.

### Significance Testing for Overlaps between Transcripts and Genomic Feature Sets

Statistical significance for overlaps between genomic feature sets (i.e., Exoniphy predicted coding exons [47], RNAz [49], and EvoFold [48] conserved RNA structures, DNase I hypersensitivity sites generated by the UW ENCODE group [54], and enhancer sets [65],[66]) and exons in transcript units or significant seqfrags in trimmed intergenic regions was calculated by permutation analysis. In each permutation round, seqfrags or TU exons were assigned random positions within intergenic regions (for novel 5′ and 3′ exons connected to annotated genes), trimmed intergenic regions (for seqfrags in intergenic regions at least 10 kb away from genes), or introns (for novel internal exons for annotated genes, as well as exons in independent sense and antisense TUs). *p* values were defined as the proportion of times that an overlap count greater than or equal to the number of observed overlaps was found in 10,000 permutations. Coordinates of genomic feature sets were obtained from the UCSC genome browser or the original publications and mapped to the hg18 genome build using the UCSC LiftOver tool when needed.

### Accession Numbers

Affymetrix tiling array data are available at GEO (record GSE19289).

## Supporting Information

Figure S1 **Positional bias towards known genes in other genome-wide transcription datasets.** Relative enrichment of intergenic read/tag frequency near annotated genes in a variety of datasets including (A) strand-specific RNA-Seq of mouse brain PolyA+ RNA [41], (B) strand-specific RNA-Seq of mouse brain rRNA-depleted total RNA (SRX012528, NCBI short read archive), (C) Cap analysis of gene expression (CAGE) tags from 41 different human libraries [12], (D) CAGE tags from 145 different mouse libraries [12], and (E) Gene Identification Signature paired-end tags (GIS-PET) from two human cancer cell lines (MCF7 and HCT116) [42]. RNA-Seq reads and CAGE tags were mapped using Bowtie as described in the Materials and Methods section. For the GIS-PET datasets, mapped ditag positions for the hg17 version of the human genome were obtained from the original publication [42] and converted to coordinates in the hg18 assembly using the UCSC LiftOver tool (http://genome.cse.ucsc.edu/). Relative enrichment ratios of reads and tags in gene-flanking regions were calculated as described for Figure 3A and 3B.
(0.14 MB PDF)

Figure S2 **Low-coverage intergenic expression is positionally biased towards known genes.** Relative enrichment of read frequency for low-coverage transcribed regions in the pooled RNA-Seq sets as a function of the distance to 5′ and 3′ ends of annotated genes in the human (red) and mouse (green) genome. The distribution for genomic DNA-Seq reads from HeLa cells is shown as a control (gray). Low coverage regions were defined as seqfrags that were detected by only a single read in the combined human and mouse RNA-Seq sets. Relative enrichment ratios of reads and tags in gene-flanking regions were calculated as described for Figure 3A and 3B.
(0.12 MB PDF)

Figure S3 **Intergenic genomic DNA-Seq reads are approximately randomly distributed.** A sample of intergenic reads was selected from public DNA-Seq datasets (gray bars) from human sperm genomic DNA and HeLa cells [43],[44] and used to draw distribution plots analogous to Figure 5 in the main text. The number of selected DNA-Seq reads in the complete or singleton sets was equal to the number of intergenic reads in the pooled human RNA-Seq dataset. The expected random distribution is indicated by a red line.
(0.14 MB PDF)

Figure S4 **Genomic DNA normalization reduces intensity bias due to probe GC content.** (A) Affymetrix tiling array image of a mouse testis PolyA+ RNA hybridization, showing the probe signal intensity in the top half and a heatmap of the GC content of the same probes in the bottom half. Lighter shades of gray and orange correspond to higher probe intensities and GC content, respectively. (B) Running median average of probe signal intensities across mouse chromosome 18 for testes PolyA+ RNA (red) and genomic DNA (green), showing a similar baseline trend in both samples. After quantile normalization of the PolyA+ sample against genomic DNA, the non-specific baseline pattern is no longer present (blue).
(0.96 MB PDF)

Figure S5 **Effect of alignment parameters on the number of uniquely mapped reads.** Singleton 32 mer reads from 9 human tissues were mapped as either 25 mer or 32 mer, allowing for 0–2 mismatches. The number of uniquely mapped reads at each parameter combination is indicated.
(0.09 MB PDF)

Figure S6 **Overview of splice junction detection and reconstruction of gene structures.** (A) Splice junction detection by Tophat (modified from [45]). (B) Outline of the method used to merge splice junctions into gene structures. See Materials and Methods for a detailed description of this figure.
(0.11 MB PDF)

Figure S7 **Precision-recall of known splice junctions in human brain single- (A, B) and paired-end (C, D) read data.** Known junctions were defined as those that bridged any two exons of a single annotated reference transcript. The effects of three different parameters were tested: anchor size, junction read coverage, and the number of times the same junction sequence was found for different splice junctions. Numbering of points corresponding to different coverage thresholds is indicated in the top left panel and is analogous for all other lines drawn. The arrow indicates the precision-recall values for the parameter settings used in the Tophat analysis of single-end reads, before filtering junctions with low-complexity sequences.
(0.15 MB PDF)

Figure S8 **PolyA/T repeat bias in junction sequences from single-end reads.** Plots showing the percentage of junction sequences containing (A) PolyA/PolyT repeats or (B) PolyG/PolyC repeats, as a function of the repeat length. Lines represent different human RNA-Seq samples and are colored as indicated on the right.
(0.12 MB PDF)

Table S1 **Read mass statistics for all RNA-Seq samples.**
(0.05 MB PDF)

Table S2 **Transcribed genomic area for all RNA-Seq samples.**
(0.05 MB PDF)

Table S3 **Proportion of intergenic reads in 10-kb regions flanking annotated genes.**
(0.04 MB PDF)

Table S4 **Human splice junction mapping statistics.**
(0.04 MB PDF)

Table S5 **Human splice junctions bridging exons between annotated genes.**
(0.09 MB XLS)

Table S6 **(A) Overlap between genomic features and novel exons in human TUs attached to known genes.** (B) Overlap between genomic features and exons in human TUs independent from known genes.
(0.05 MB PDF)

Table S7 **Alternative splice junctions connecting to unannotated upstream promoters in the human genome.**
(0.07 MB XLS)

Table S8 **(A) Overlap between significant seqfrags in trimmed intergenic regions and genomic features.** (B) Overlap between seqfrag clusters in trimmed intergenic regions and genomic features.
(0.05 MB PDF)

Table S9 **RNA sources for tiling array experiments.**
(0.04 MB PDF)

## Abbreviations

APA: alternative cleavage and polyadenylation
BW: bandwidth parameter
CAGE: capped analysis of gene expression
CNV: copy number variation
lincRNAs: large intervening noncoding RNAs
ncRNAs: noncoding RNAs
ORF: open reading frames
pasRNA: promoter-associated RNA
TSS: transcription start site
TTS: transcription termination site
TU: transcript unit
TUF: transcript of unknown function
