# Supplementary figures and images for: Most “Dark Matter” Transcripts Are Associated With Known Genes
Source: PLoS Biol. 2010 May 18;8(5):e1000371. doi: 10.1371/journal.pbio.1000371 (PMC2872640; doi:10.1371/journal.pbio.1000371)

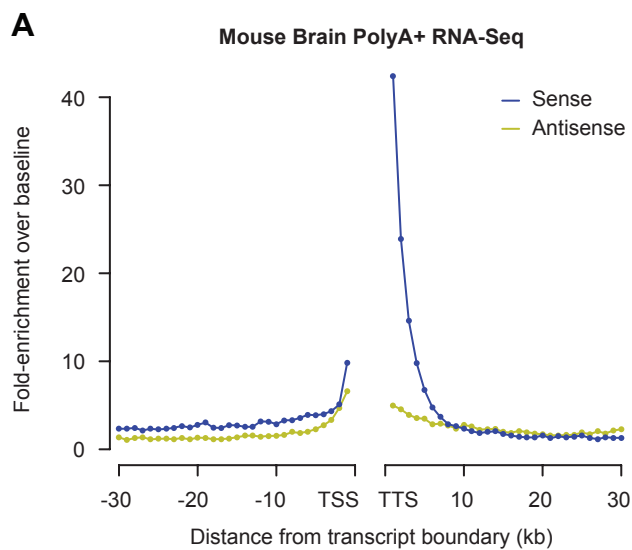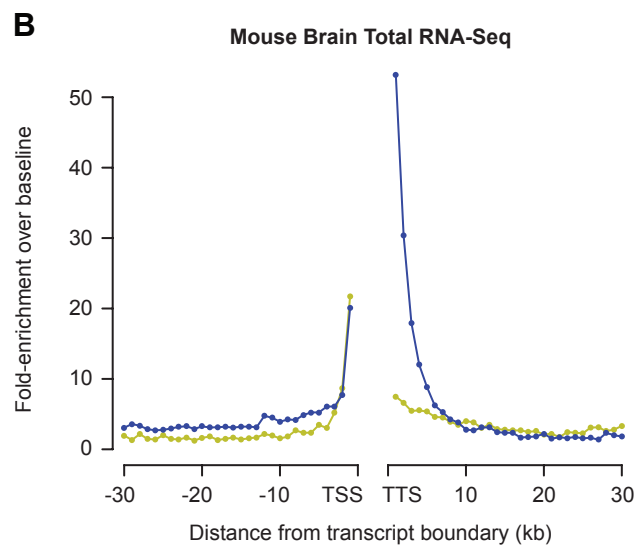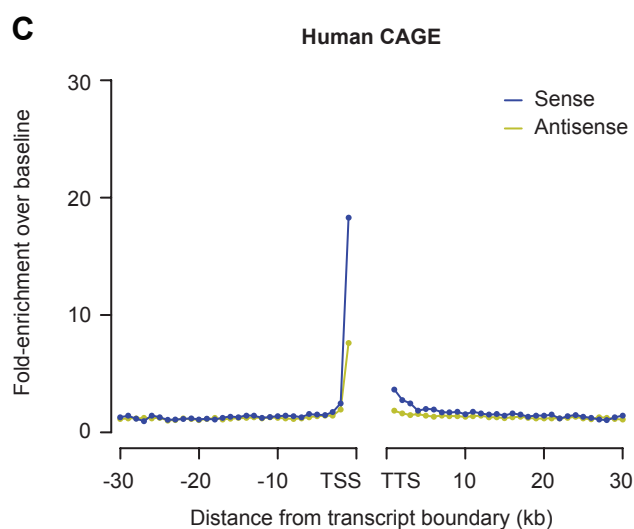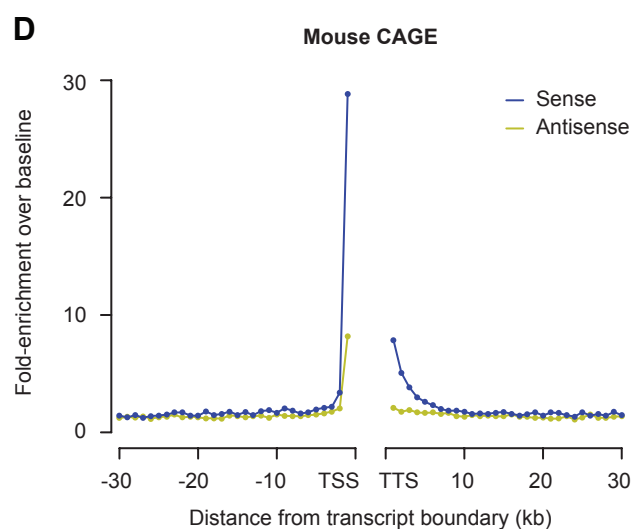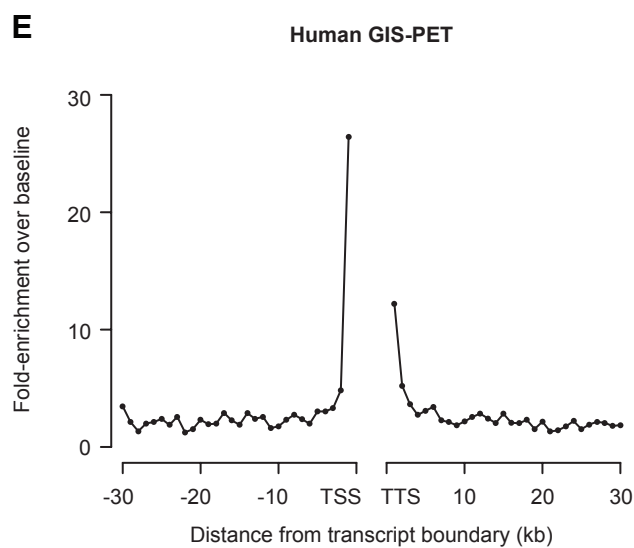

Supplement: Figure S1 — Positional bias towards known genes in other genome-wide transcription datasets. Relative enrichment of intergenic read/tag frequency near annotated genes in a variety of datasets including (A) strand-specific RNA-Seq of mouse brain PolyA+ RNA [41], (B) strand-specific RNA-Seq of mouse brain rRNA-depleted total RNA (SRX012528, NCBI short read archive), (C) Cap analysis of gene expression (CAGE) tags from 41 different human libraries [12], (D) CAGE tags from 145 different mouse libraries [12], and (E) Gene Identification Signature paired-end tags (GIS-PET) from two human cancer cell lines (MCF7 and HCT116) [42]. RNA-Seq reads and CAGE tags were mapped using Bowtie as described in the Materials and Methods section. For the GIS-PET datasets, mapped ditag positions for the hg17 version of the human genome were obtained from the original publication [42] and converted to coordinates in the hg18 assembly using the UCSC LiftOver tool (http://genome.cse.ucsc.edu/). Relative enrichment ratios of reads and tags in gene-flanking regions were calculated as described for Figure 3A and 3B. (0.14 MB PDF) [file pbio.1000371.s001.pdf]

Singleton Human and Mouse PolyA+ RNA-Seq

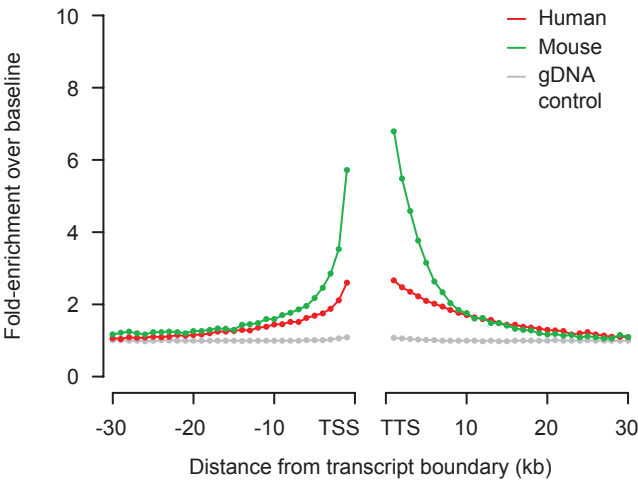

Supplement: Figure S2 — Low-coverage intergenic expression is positionally biased towards known genes. Relative enrichment of read frequency for low-coverage transcribed regions in the pooled RNA-Seq sets as a function of the distance to 5′ and 3′ ends of annotated genes in the human (red) and mouse (green) genome. The distribution for genomic DNA-Seq reads from HeLa cells is shown as a control (gray). Low coverage regions were defined as seqfrags that were detected by only a single read in the combined human and mouse RNA-Seq sets. Relative enrichment ratios of reads and tags in gene-flanking regions were calculated as described for Figure 3A and 3B. (0.12 MB PDF) [file pbio.1000371.s002.pdf]

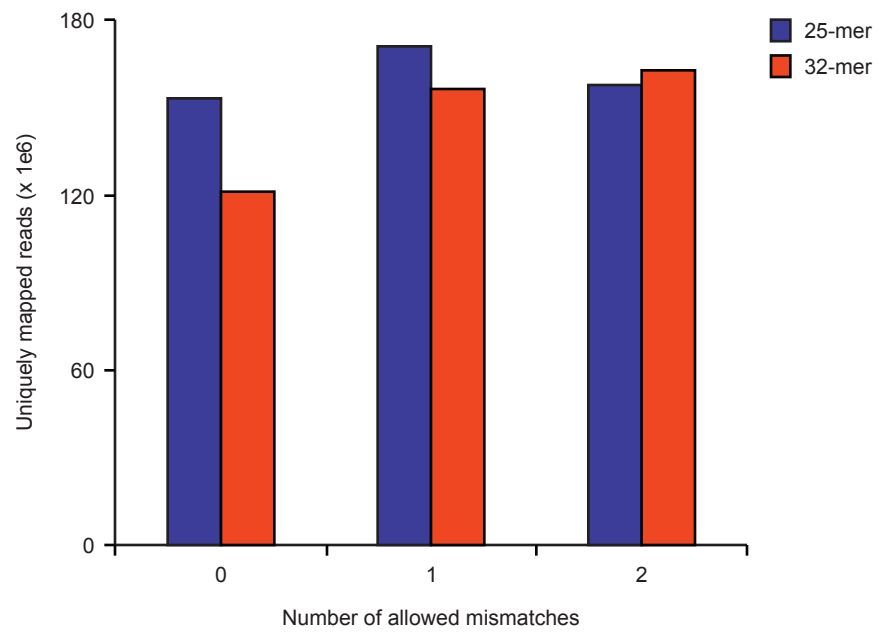

Supplement: Figure S5 — Effect of alignment parameters on the number of uniquely mapped reads. Singleton 32 mer reads from 9 human tissues were mapped as either 25 mer or 32 mer, allowing for 0–2 mismatches. The number of uniquely mapped reads at each parameter combination is indicated. (0.09 MB PDF) [file pbio.1000371.s005.pdf]

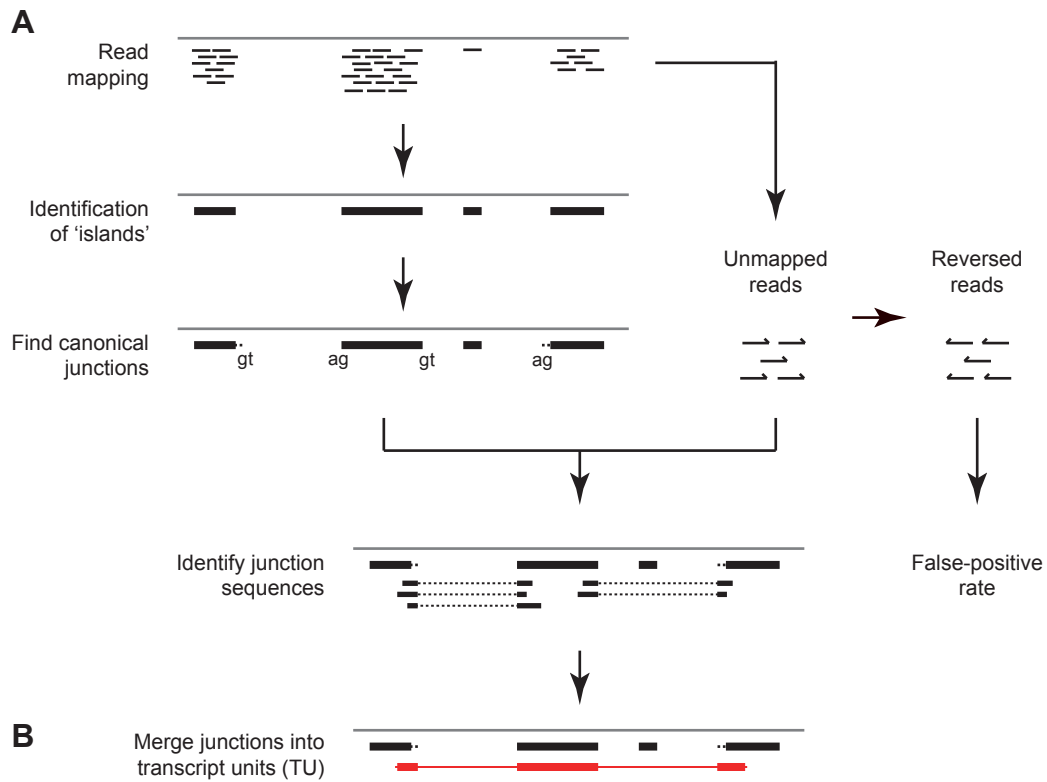

Supplement: Figure S6 — Overview of splice junction detection and reconstruction of gene structures. (A) Splice junction detection by Tophat (modified from [45]). (B) Outline of the method used to merge splice junctions into gene structures. See Materials and Methods for a detailed description of this figure. (0.11 MB PDF) [file pbio.1000371.s006.pdf]
