# Supplementary material for: Most “Dark Matter” Transcripts Are Associated With Known Genes
Source: PLoS Biol. 2010 May 18;8(5):e1000371. doi: 10.1371/journal.pbio.1000371 (PMC2872640; doi:10.1371/journal.pbio.1000371)
Supplement: Figure S3 — Intergenic genomic DNA-Seq reads are approximately randomly distributed. A sample of intergenic reads was selected from public DNA-Seq datasets (gray bars) from human sperm genomic DNA and HeLa cells [43],[44] and used to draw distribution plots analogous to Figure 5 in the main text. The number of selected DNA-Seq reads in the complete or singleton sets was equal to the number of intergenic reads in the pooled human RNA-Seq dataset. The expected random distribution is indicated by a red line. (0.14 MB PDF) [file pbio.1000371.s003.pdf]

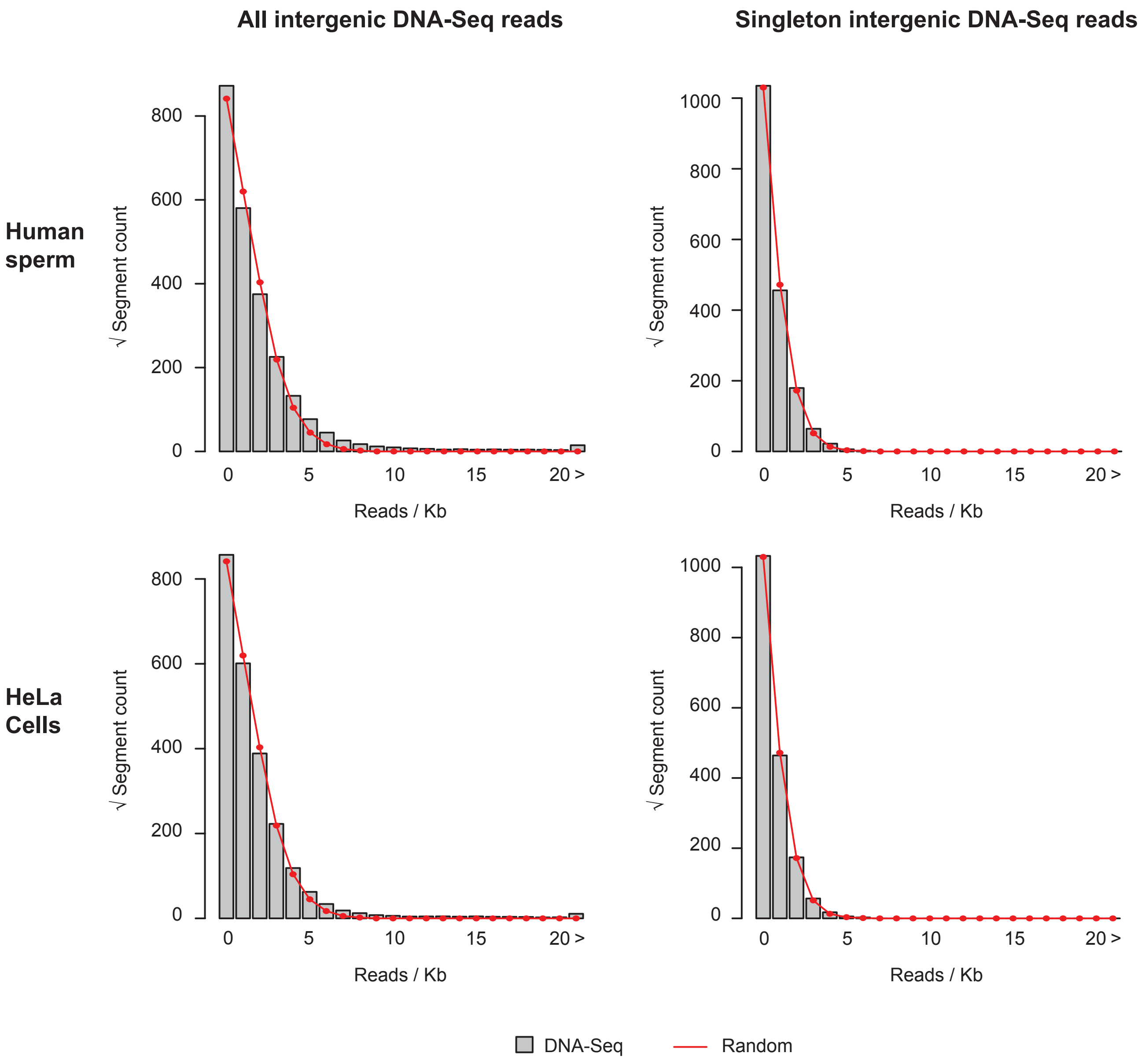

All intergenic DNA-Seq reads
Singleton intergenic DNA-Seq reads
Human sperm
HeLa Cells
√ Segment count
Reads / Kb
DNA-Seq
Random
