# Supplementary material for: Most “Dark Matter” Transcripts Are Associated With Known Genes
Source: PLoS Biol. 2010 May 18;8(5):e1000371. doi: 10.1371/journal.pbio.1000371 (PMC2872640; doi:10.1371/journal.pbio.1000371)
Supplement: Figure S4 — Genomic DNA normalization reduces intensity bias due to probe GC content. (A) Affymetrix tiling array image of a mouse testis PolyA+ RNA hybridization, showing the probe signal intensity in the top half and a heatmap of the GC content of the same probes in the bottom half. Lighter shades of gray and orange correspond to higher probe intensities and GC content, respectively. (B) Running median average of probe signal intensities across mouse chromosome 18 for testes PolyA+ RNA (red) and genomic DNA (green), showing a similar baseline trend in both samples. After quantile normalization of the PolyA+ sample against genomic DNA, the non-specific baseline pattern is no longer present (blue). (0.96 MB PDF) [file pbio.1000371.s004.pdf]

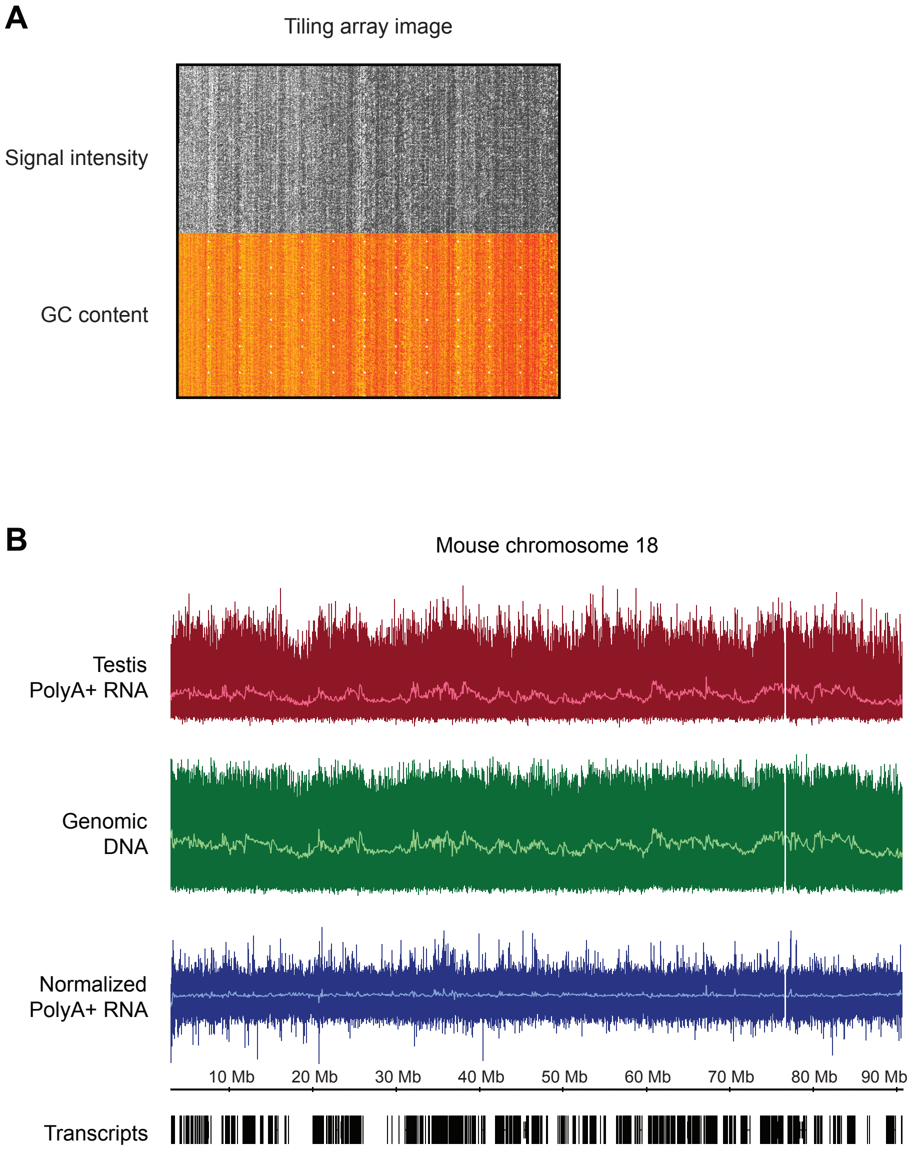
A
Tiling array image
Signal intensity
GC content
B
Mouse chromosome 18
Testis
PolyA+ RNA
Genomic
DNA
Normalized
PolyA+ RNA
10 Mb
20 Mb
30 Mb
40 Mb
50 Mb
60 Mb
70 Mb
80 Mb
90 Mb
Transcripts
