# Supplementary material for: Most “Dark Matter” Transcripts Are Associated With Known Genes
Source: PLoS Biol. 2010 May 18;8(5):e1000371. doi: 10.1371/journal.pbio.1000371 (PMC2872640; doi:10.1371/journal.pbio.1000371)
Supplement: Figure S7 — Precision-recall of known splice junctions in human brain single- (A, B) and paired-end (C, D) read data. Known junctions were defined as those that bridged any two exons of a single annotated reference transcript. The effects of three different parameters were tested: anchor size, junction read coverage, and the number of times the same junction sequence was found for different splice junctions. Numbering of points corresponding to different coverage thresholds is indicated in the top left panel and is analogous for all other lines drawn. The arrow indicates the precision-recall values for the parameter settings used in the Tophat analysis of single-end reads, before filtering junctions with low-complexity sequences. (0.15 MB PDF) [file pbio.1000371.s007.pdf]

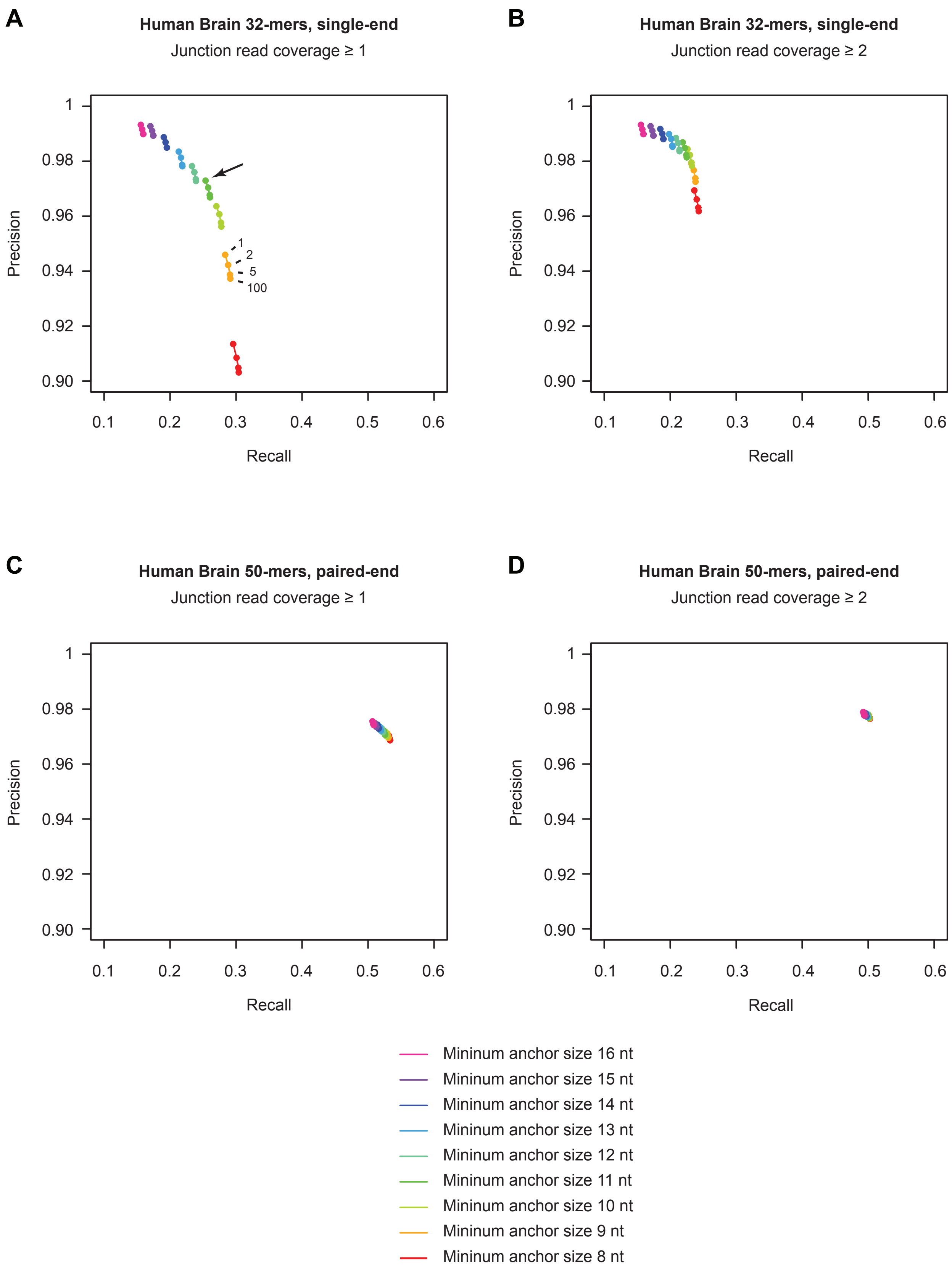
A
Human Brain 32-mers, single-end
Junction read coverage ≥ 1
B
Human Brain 32-mers, single-end
Junction read coverage ≥ 2
C
Human Brain 50-mers, paired-end
Junction read coverage ≥ 1
D
Human Brain 50-mers, paired-end
Junction read coverage ≥ 2
Precision
Recall
1
0.98
0.96
0.94
0.92
0.90
0.1
0.2
0.3
0.4
0.5
0.6
1
2
5
100
Mininum anchor size 16 nt
Mininum anchor size 15 nt
Mininum anchor size 14 nt
Mininum anchor size 13 nt
Mininum anchor size 12 nt
Mininum anchor size 11 nt
Mininum anchor size 10 nt
Mininum anchor size 9 nt
Mininum anchor size 8 nt
