# Supplementary material for: Most “Dark Matter” Transcripts Are Associated With Known Genes
Source: PLoS Biol. 2010 May 18;8(5):e1000371. doi: 10.1371/journal.pbio.1000371 (PMC2872640; doi:10.1371/journal.pbio.1000371)
Supplement: Table S1 — Read mass statistics for all RNA-Seq samples. (0.05 MB PDF) [file pbio.1000371.s009.pdf]

Table S1. Read mass statistics for all RNA-Seq samples

Human

	Total reads	Mapped – all	%	Mapped – unique	%	G exon	%	G+M exon	%	G+M+E exon	%	G full	%	G+M full	%	G+M+E full	%	SF-intron	SF-IG	% SF IG
**Adipose**	27,752,231	23,718,988	85%	17,617,327	63%	16,145,945	92%	16,432,843	93%	16,553,137	94%	17,075,382	97%	17,218,859	98%	17,312,409	98%	929,437	541,945	37%
**Brain_HCT168**	17,246,957	14,683,920	85%	10,384,897	60%	9,040,178	87%	9,308,526	90%	9,388,918	90%	9,780,039	94%	9,983,292	96%	10,068,778	97%	739,861	604,858	45%
**Brain_s1368**	10,112,968	7,823,077	77%	5,547,979	55%	4,338,775	78%	4,515,228	81%	4,577,206	83%	5,165,326	93%	5,276,449	95%	5,350,203	96%	826,551	382,653	32%
**Colon**	28,435,996	24,181,748	85%	16,953,478	60%	15,384,593	91%	15,704,498	93%	15,837,542	93%	16,315,580	96%	16,488,837	97%	16,582,287	98%	930,987	637,898	41%
**Heart**	20,169,301	17,081,466	85%	10,836,393	54%	9,537,938	88%	9,730,009	90%	9,820,594	91%	10,302,356	95%	10,400,746	96%	10,450,454	96%	764,418	534,037	41%
**Liver**	18,517,121	16,129,228	87%	11,402,292	62%	10,602,876	93%	10,741,089	94%	10,798,601	95%	11,082,822	97%	11,161,653	98%	11,221,898	98%	479,946	319,470	40%
**Lymphnode**	27,492,254	24,186,880	88%	15,761,345	57%	13,718,706	87%	14,183,657	90%	14,399,945	91%	15,268,841	97%	15,427,764	98%	15,540,196	99%	1,550,135	492,504	24%
**Skeletal Muscle**	22,640,454	19,530,861	86%	14,075,679	62%	12,899,206	92%	13,116,432	93%	13,184,789	94%	13,583,035	97%	13,717,681	97%	13,784,182	98%	683,829	492,644	42%
**Testes**	27,303,938	24,067,201	88%	18,360,524	67%	16,299,444	89%	16,658,080	91%	16,888,245	92%	17,500,470	95%	17,737,793	97%	17,949,885	98%	1,201,026	860,054	42%
**Brain**	46,823,666	38,864,040	83%	34,224,865	73%	29,151,132	85%	30,190,430	88%	30,559,507	89%	32,521,636	95%	33,108,512	97%	33,448,226	98%	3,396,746	1,703,229	33%
**UHR**	45,111,090	37,941,778	84%	30,413,555	67%	26,205,879	86%	27,015,317	89%	27,382,282	90%	29,172,495	96%	29,553,966	97%	29,845,217	98%	3,016,339	1,241,060	29%
**Totals**	291,605,976	248,209,187	85%	185,578,334	64%	163,324,672	88%	167,596,109	90%	169,390,766	91%	177,767,982	96%	180,075,552	97%	181,553,735	98%	14,519,275	7,810,352	35%

Mouse

	Total reads	Mapped – all	%	Mapped – unique	%	G exon	%	G+M exon	%	G+M+E exon	%	G full	%	G+M full	%	G+M+E full	%	SF-intron	SF-IG	% SF IG
**Brain**	78,898,555	48,912,609	62%	35,088,405	44%	31,069,233	89%	32,412,957	92%	32,601,100	93%	32,635,321	93%	34,009,680	97%	34,166,074	97%	1,566,088	2,453,084	61%
**Liver**	71,751,153	36,522,941	51%	23,601,947	33%	21,174,977	90%	22,029,688	93%	22,100,560	94%	22,154,200	94%	23,038,600	98%	23,110,343	98%	979,223	1,447,747	60%
**Skeletal Muscle**	69,770,965	40,242,380	58%	27,028,806	39%	23,254,497	86%	24,897,729	92%	24,987,573	92%	24,443,100	90%	26,185,140	97%	26,297,234	97%	1,188,603	2,585,706	69%
**Totals**	220,420,673	125,677,930	57%	85,719,158	39%	75,498,707	88%	79,340,374	93%	79,689,233	93%	79,232,621	92%	83,233,420	97%	83,573,651	97%	3,733,914	6,486,537	63%

G – Kown gene annotations derived from UCSC, ENSEMBL and NCBI RefSeq
M – UCSC mRNA track
E – UCSC spliced EST track
SF – Seqfrag
IG – Intergenic
