# Supplementary material for: Most “Dark Matter” Transcripts Are Associated With Known Genes
Source: PLoS Biol. 2010 May 18;8(5):e1000371. doi: 10.1371/journal.pbio.1000371 (PMC2872640; doi:10.1371/journal.pbio.1000371)
Supplement: Table S2 — Transcribed genomic area for all RNA-Seq samples. (0.05 MB PDF) [file pbio.1000371.s010.pdf]

Table S2. Transcribed genomic area for all RNA-Seq samples

Human

	Area (bp)	%	G exon (bp)	%	G+M exon (bp)	%	G+M+E exon (bp)	%	G full (bp)	%	G+M full (bp)	%	G+M+E full (bp)	%	SF intron (bp)	SF IG (bp)	% SF IG
**Adipose**	41,411,320	1%	25,676,370	62%	27,787,569	67%	28,922,236	70%	37,545,298	91%	38,668,769	93%	39,705,168	96%	11,868,928	3,866,022	25%
**Brain_HCT168**	40,437,509	1%	25,788,391	64%	27,824,373	69%	28,571,355	71%	35,257,683	87%	36,792,473	91%	38,091,033	94%	9,469,292	5,179,826	35%
**Brain_s1368**	43,366,584	1%	22,346,454	52%	24,657,115	57%	25,553,956	59%	37,997,808	88%	39,468,367	91%	40,913,819	94%	15,651,354	5,368,776	26%
**Colon**	40,320,530	1%	25,767,789	64%	27,850,120	69%	28,951,459	72%	36,511,691	91%	37,673,768	93%	38,696,238	96%	10,743,902	3,808,839	26%
**Heart**	29,813,425	1%	20,888,192	70%	22,208,565	74%	22,847,189	77%	27,159,186	91%	27,978,616	94%	28,638,187	96%	6,270,994	2,654,239	30%
**Liver**	26,738,880	1%	17,957,103	67%	19,190,740	72%	19,777,403	74%	24,088,257	90%	24,861,971	93%	25,526,212	95%	6,131,154	2,650,623	30%
**Lymphnode**	52,963,430	2%	28,360,032	54%	31,870,112	60%	33,738,468	64%	47,576,881	90%	49,127,571	93%	50,622,942	96%	19,216,849	5,386,549	22%
**Skeletal Muscle**	29,798,906	1%	21,489,016	72%	22,748,874	76%	23,317,203	78%	26,865,969	90%	27,766,951	93%	28,466,379	96%	5,376,953	2,932,937	35%
**Testes**	62,843,913	2%	34,704,927	55%	37,950,793	60%	40,269,375	64%	52,906,864	84%	55,281,426	88%	58,212,473	93%	18,201,937	9,937,049	35%
**Paired Brain**	153,436,585	5%	50,572,412	33%	60,679,240	40%	65,479,198	43%	126,912,216	83%	133,229,934	87%	140,509,651	92%	76,339,804	26,524,369	26%
**Paired UHR**	147,388,269	5%	49,744,512	34%	59,315,619	40%	64,473,411	44%	124,528,360	84%	129,988,133	88%	136,926,627	93%	74,783,848	22,859,909	23%
**Totals**	293,887,303	10%	65,409,635	22%	81,730,831	28%	90,780,713	31%	234,858,385	80%	246,475,629	84%	263,937,044	90%	169,448,750	59,028,918	26%

Mouse

	Area (bp)	%	G exon (bp)	%	G+M exon (bp)	%	G+M+E exon (bp)	%	G full (bp)	%	G+M full (bp)	%	G+M+E full (bp)	%	SF intron (bp)	SF IG (bp)	% SF IG
**Brain**	66,975,941	2%	40,676,397	61%	45,604,061	68%	47,114,116	70%	56,631,658	85%	60,257,017	90%	61,445,373	92%	15,955,261	10,344,283	39%
**Liver**	42,637,366	2%	26,252,904	62%	28,883,928	68%	29,560,422	69%	37,283,275	87%	39,121,080	92%	39,813,987	93%	11,030,371	5,354,091	33%
**Skeletal Muscle**	38,490,216	1%	28,001,792	73%	30,213,529	78%	30,850,111	80%	33,549,775	87%	35,304,344	92%	35,832,840	93%	5,547,983	4,940,441	47%
**Totals**	92,293,245	3%	46,686,330	51%	53,761,164	58%	55,887,494	61%	75,946,761	82%	80,993,813	88%	82,977,849	90%	29,260,431	16,346,484	36%

G – Kown gene annotations derived from UCSC, ENSEMBL and NCBI RefSeq
M – UCSC mRNA track
E – UCSC spliced EST track
SF – Seqfrag
IG – Intergenic
