# Supplementary material for: Most “Dark Matter” Transcripts Are Associated With Known Genes
Source: PLoS Biol. 2010 May 18;8(5):e1000371. doi: 10.1371/journal.pbio.1000371 (PMC2872640; doi:10.1371/journal.pbio.1000371)
Supplement: Table S3 — Proportion of intergenic reads in 10-kb regions flanking annotated genes. (0.04 MB PDF) [file pbio.1000371.s011.pdf]

Table S3. Proportion of intergenic reads in 10kb regions flanking annotated genes

Sample type	% IG reads[1)]	% IGF reads[2)]	Outlier seqfrags[3)]	% IGF reads – no outliers [4)]	References [5)]
Human PolA+ RNA (9 tissues)	4.2%	80.4%	43 (13 kb)	80.7%	[16, this paper]
Mouse PolA+ RNA (3 tissues)	7.6%	78.4%	32 (8 kb)	84.8%	[36]
Mouse PolA+, strand-specific (1 tissue)	10.3%	70.2%	5 (2 kb)	70.8%	[41]
Human rRNA depleted, strand-specific (2 samples)	9.7%	52.7%	41 (14 kb)	51.6%	[35]
Mouse rRNA depleted, strand-specific (1 tissue)	27.0%	30.7%	21 (5 kb)	71.1%	SRX012528
Human CAGE (145 libraries)	17.0%	72.4%	19 (4 kb)	72.0%	[12]
Mouse CAGE (41 libraries)	17.0%	59.3%	7 (1 kb)	70.0%	[12]
Human GIS-PET (2 cell lines)	4.5%	76.5%	0 (0 kb)	76.5%	[42]

[1)] Percentage of reads in intergenic (IG) regions

[2)] Percentage of intergenic gene-flanking (IGF) reads in 10kb intergenic regions directly adjacent to annotated genes

[3)] Number of seqfrags in intergenic regions with excessive read counts (>10,000). Genomic area covered by outlier seqfrags is indicated between brackets.

[4)] Percentage of intergenic reads in 10kb gene-flanking regions after removal of outlier seqfrags

[5)] Reference to the source of the data set(s) used. Mouse brain total RNA-Seq data were obtained from the NCBI short read archive (SRX012528)
