# Supplementary material for: Most “Dark Matter” Transcripts Are Associated With Known Genes
Source: PLoS Biol. 2010 May 18;8(5):e1000371. doi: 10.1371/journal.pbio.1000371 (PMC2872640; doi:10.1371/journal.pbio.1000371)
Supplement: Table S4 — Human splice junction mapping statistics. (0.04 MB PDF) [file pbio.1000371.s012.pdf]

Table S4. Human splice junction mapping statistics

Tissue	# Junctions	# Known	% Known	# Alternative	% Alternative	Novel
Single Adipose	40,569	40,021	98.6%	860	2.1%	548
Single Brain_HCT168	30,287	29,924	98.8%	466	1.5%	363
Single Brain_s1368	21,448	21,138	98.6%	288	1.3%	310
Single Colon	35,995	35,471	98.5%	792	2.2%	524
Single Heart	26,808	26,482	98.8%	459	1.7%	326
Single Liver	21,284	21,039	98.8%	292	1.4%	245
Single Lymph Node	39,857	39,151	98.2%	1,033	2.6%	706
Single skelMuscle	32,731	32,344	98.8%	595	1.8%	387
Single Testes	55,843	54,024	96.7%	1,580	2.8%	1,819
Paired Brain	114,400	111,004	97.0%	4,564	4.0%	3,396
Paired UHR	108,913	106,314	97.6%	3,596	3.3%	2,599
**Total**	**528,135**	**516,912**	**97.9%**	**14,525**	**2.8%**	**11,223**
**Total unique**	**160,516**	**151,708**	**94.5%**	**16,809**	**10.5%**	**8,808**
**Total known**		**257,257**	**59.0%**			
