# Supplementary material for: Most “Dark Matter” Transcripts Are Associated With Known Genes
Source: PLoS Biol. 2010 May 18;8(5):e1000371. doi: 10.1371/journal.pbio.1000371 (PMC2872640; doi:10.1371/journal.pbio.1000371)
Supplement: Table S6 — (A) Overlap between genomic features and novel exons in human TUs attached to known genes. (B) Overlap between genomic features and exons in human TUs independent from known genes. (0.05 MB PDF) [file pbio.1000371.s014.pdf]

Table S6A. Overlap between genomic features and novel exons in human TUs attached to known genes

Feature set	Features	TU exons	Expected overlap[1)]		Observed overlap		P-value [2)]	References
**5' TU exons**								
Evofold	47,510	723	0	(0.0%)	5	(0.7%)	0.0004	[7]
RNAz	35,985	723	1	(0.1%)	7	(1.0%)	0.0133	[8]
Exoniphy	178,162	723	0	(0.0%)	137	(18.9%)	< 0.0001	[9]
DNaseI hotspots, pooled [3)]	1,168,817	723	72	9.96%	519	(71.8%)	< 0.0001	[10]
DNaseI hotspots, replicated [4)]	473,232	723	43	5.95%	459	(63.5%)	< 0.0001	[10]
**Internal TU exons**								
Evofold	47,510	3,451	3	(0.1%)	30	(0.9%)	< 0.0001	[7]
RNAz	35,985	3,451	8	(0.2%)	54	(1.6%)	< 0.0001	[8]
Exoniphy	178,162	3,451	8	(0.2%)	232	(6.7%)	< 0.0001	[9]
**3' TU exons**								
Evofold	47,510	370	0	(0.0%)	1	(0.0%)	0.09	[7]
RNAz	35,985	370	0	(0.0%)	5	(1.4%)	0.0006	[8]
Exoniphy	178,162	370	0	(0.0%)	76	(20.5%)	< 0.0001	[9]

Table S6B. Overlap between genomic features and exons in human TUs independent from known genes

Feature set	Features	TU exons	Expected overlap[1)]		Observed overlap		P-value [2)]	References
**Intergenic TU exons**								
Evofold	47,510	2,821	2	(0.0%)	11	(0.0%)	< 0.0001	[7]
RNAz	35,985	2,821	5	(0.2%)	17	(0.6%)	< 0.0001	[8]
Exoniphy	178,162	2,821	0	(0.0%)	297	(10.5%)	< 0.0001	[9]
DNaseI hotspots, pooled [3)]	1,168,817	2,821	267	(9.5%)	1,194	(42.3%)	< 0.0001	[10]
DNaseI hotspots, replicated [4)]	473,232	2,821	164	(5.8%)	945	(33.5%)	< 0.0001	[10]
**Exons in TUs overlapping genes, sense strand**								
Evofold	47,510	1,069	1	(0.1%)	3	(0.3%)	0.07	[7]
RNAz	35,985	1,069	2	(0.2%)	11	(1.0%)	< 0.0001	[8]
Exoniphy	178,162	1,069	2	(0.2%)	171	(16.0%)	< 0.0001	[9]
**Exons in TUs overlapping genes, antisense strand**								
Evofold	47,510	2,295	2	(0.1%)	11	(0.5%)	< 0.0001	[7]
RNAz	35,985	2,295	5	(0.2%)	23	(1.0%)	< 0.0001	[8]
Exoniphy	178,162	2,295	5	(0.2%)	483	(21.0%)	< 0.0001	[9]

[1)] Median of the number of seqfrags or seqfrag clusters overlapping with set features in 10,000 permutations, where the positions of seqfrags in intergenic regions were randomized. The percentage of seqfrags or seqfrag clusters that overlap features is indicated between brackets.

[2)] P-value for observed overlap based on the overlap counts in 10,000 randomized permutations

[3)] Aggregate of DNaseI hypersensitive zones identified by the HotSpot algorithm [12] in all replicates of 11 cell lines (BJ, Caco-2, GM06990, HepG2, HL-60, HUVEC, K562, SK-N-SH_RA, SKMC, Th1, Th2). These data sets were generated by the UW ENCODE group.

[4)] Aggregate of DNaseI hypersensitive zones identified by the HotSpot algorithm in 8 cell lines (BJ, Caco-2, GM06990, HepG2, HL-60, K562, SK-N-SH_RA, SKMC), selecting only hypersensitive zones that were found in both replicate samples for each cell line. These data sets were generated by the UW ENCODE group.
