# Supplementary material for: Most “Dark Matter” Transcripts Are Associated With Known Genes
Source: PLoS Biol. 2010 May 18;8(5):e1000371. doi: 10.1371/journal.pbio.1000371 (PMC2872640; doi:10.1371/journal.pbio.1000371)
Supplement: Table S8 — (A) Overlap between significant seqfrags in trimmed intergenic regions and genomic features. (B) Overlap between seqfrag clusters in trimmed intergenic regions and genomic features. (0.05 MB PDF) [file pbio.1000371.s016.pdf]

Table S8A. Overlap between significant seqfrags in trimmed intergenic regions and genomic features

Feature set	Features	Seqfrags	Expected overlap[1)]		Observed overlap		P-value [2)]	References
**Human**								
Evofold	47,510	15,646	25	(0.2%)	27	(0.2%)	0.41	[48]
RNAz	35,985	15,646	43	(0.3%)	128	(0.8%)	< 0.0001	[49]
Exoniphy	178,162	15,646	6	(0.0%)	157	(1.0%)	< 0.0001	[47]
DNaseI hotspots, pooled [3)]	1,168,817	15,646	1,886	(12.1%)	6,407	(40.9%)	< 0.0001	[54]
DNaseI hotspots, replicated [4)]	473,232	15,646	1,075	(6.9%)	4,268	(27.3%)	< 0.0001	[54]
Heart enhancers	41,930	15,646	118	(0.8%)	158	(1.0%)	0.0005	[65]
**Mouse**								
Exoniphy	172,859	11,533	2	(0.0%)	261	(2.3%)	< 0.0001	[47]
lincRNAs	2,127	11,533	4	(0.0%)	95	(0.8%)	< 0.0001	[19]
p300 enhancers	4,686	11,533	13	(0.1%)	33	(0.3%)	< 0.0001	[66]

Table S8B. Overlap between seqfrag clusters in trimmed intergenic regions and genomic features

Feature set	Features	Clusters	Expected overlap[1)]		Observed overlap		P-value [2)]	References
**Human**								
Evofold	47,510	7,976	25	(0.3%)	25	(0.3%)	0.11	[48]
RNAz	35,985	7,976	37	(0.5%)	99	(1.2%)	< 0.0001	[49]
Exoniphy	178,162	7,976	5	(0.1%)	114	(1.4%)	< 0.0001	[47]
DNaseI hotspots, pooled [3)]	1,168,817	7,976	1,215	(15.2%)	3,528	(44.2%)	< 0.0001	[54]
DNaseI hotspots, replicated [4)]	473,232	7,976	675	(8.5%)	2,320	(29.1%)	< 0.0001	[54]
Heart enhancers	41,930	7,976	79	(1.0%)	103	(1.3%)	0.0050	[65]
**Mouse**								
Exoniphy	172,859	5,506	2	(0.0%)	192	(3.5%)	< 0.0001	[47]
lincRNAs	2,127	5,506	2	(0.0%)	31	(0.6%)	< 0.0001	[19]
p300 enhancers	4,686	5,506	7	(0.1%)	16	(0.3%)	0.0052	[66]

[1)] Median of the number of seqfrags or seqfrag clusters overlapping with set features in 10,000 permutations, where the positions of seqfrags in intergenic regions were randomized. The percentage of seqfrags or seqfrag clusters that overlap features is indicated between brackets.

[2)] P-value for observed overlap based on the overlap counts in 10,000 randomized permutations

[3)] Aggregate of DNaseI hypersensitive zones identified by the HotSpot algorithm [14] in all replicates of 11 cell lines (BJ, Caco-2, GM06990, HepG2, HL-60, HUVEC, K562, SK-N-SH_RA, SKMC, Th1, Th2). These data sets were generated by the UW ENCODE group.

[4)] Aggregate of DNaseI hypersensitive zones identified by the HotSpot algorithm in 8 cell lines (BJ, Caco-2, GM06990, HepG2, HL-60, K562, SK-N-SH_RA, SKMC), selecting only hypersensitive zones that were found in both replicate samples for each cell line. These data sets were generated by the UW ENCODE group.
