# Supplementary material for: Most “Dark Matter” Transcripts Are Associated With Known Genes
Source: PLoS Biol. 2010 May 18;8(5):e1000371. doi: 10.1371/journal.pbio.1000371 (PMC2872640; doi:10.1371/journal.pbio.1000371)
Supplement: Table S9 — RNA sources for tiling array experiments. (0.04 MB PDF) [file pbio.1000371.s017.pdf]

Table S9. RNA sources for tiling array experiments

Sample type	Supplier	Catalog No	Lot No	Description
Human Brain Poly A+ RNA	Clontech	636102	6120217	Whole brain pooled from 8 Caucasian males, ages 43-66; cause of death: sudden death
Human Heart Poly A+ RNA	Clontech	636113	6060184	Normal human heart pooled from 3 Caucasian males, ages 33, 55 and 55; cause of death: trauma
Human Liver Poly A+ RNA	Clontech	636101	6100188	Normal, whole liver pooled from 1 male Caucasian, age 35; cause of death: sudden death
Human Testis Poly A+ RNA	Clontech	636115	6090242	Normal, human testis pooled from 45 male Caucasians, ages 14-64 years old; cause of death: sudden death
Human Brain Total RNA	Clontech	636530	6120234	Normal, whole brains from 2 male Caucasians, ages 47-55; cause of death: sudden death
Human Heart Total RNA	Clontech	636532	7030456	Normal human heart pooled from 3 male/female Caucasians; ages 20-42; cause of death: trauma
Human Liver Total RNA	Clontech	636531	7030174	Normal liver pooled from a 51-yr-old male Caucasian; cause of death: sudden death
Human Testis Total RNA	Clontech	636533	7010255	Normal human testis pooled from 39 Caucasians, ages 14-64; cause of death: sudden death
Mouse Brain Poly A+ RNA	Clontech	6616-1	4020586	Normal, whole brains pooled from 600 male/female BALB/c mice, age 8-12 weeks
Mouse Heart Poly A+ RNA	Clontech	6611-1	3030999	Normal hearts pooled from 200 male/female BALB/c mice, ages: 8-12 weeks
Mouse Heart Poly A+ RNA	Clontech	636202	7060284	Normal whole hearts pooled from 1600 female BALB/c mice; ages 8-12 weeks
Mouse Liver Poly A+ RNA	Clontech	636201	4020159	Normal mouse livers pooled from >300 male/female BALB/c mice, ages 6-12 weeks
Mouse Testis Poly A+ RNA	Clontech	636203	5010515	Normal mouse testis pooled from 200 male BALB/c mice, ages 8-12 weeks
Mouse Brain Total RNA	Clontech	636601	6100187	Normal, whole brains pooled from 600 BALB/c mice, ages 9-12 weeks
Mouse Liver Total RNA	Clontech	636603	5110355	Normal, whole liver pooled from 800 male BALB/c mice, ages: 9-12 weeks
Mouse Testis Total RNA	Clontech	636606	7040109	Normal testis pooled from 200 male BALB/c mice; ages 6-10 weeks
Mouse Heart Total RNA	Clontech	636602	7050138	Normal whole hearts pooled from 200 BALB/c male/female mice, ages: 8-12 weeks
