# Supplementary material for: Most “Dark Matter” Transcripts Are Associated With Known Genes
Source: PLoS Biol. 2010 May 18;8(5):e1000371. doi: 10.1371/journal.pbio.1000371 (PMC2872640; doi:10.1371/journal.pbio.1000371)
Supplement: Figure S8 — PolyA/T repeat bias in junction sequences from single-end reads. Plots showing the percentage of junction sequences containing (A) PolyA/PolyT repeats or (B) PolyG/PolyC repeats, as a function of the repeat length. Lines represent different human RNA-Seq samples and are colored as indicated on the right. (0.12 MB PDF) [file pbio.1000371.s008.pdf]

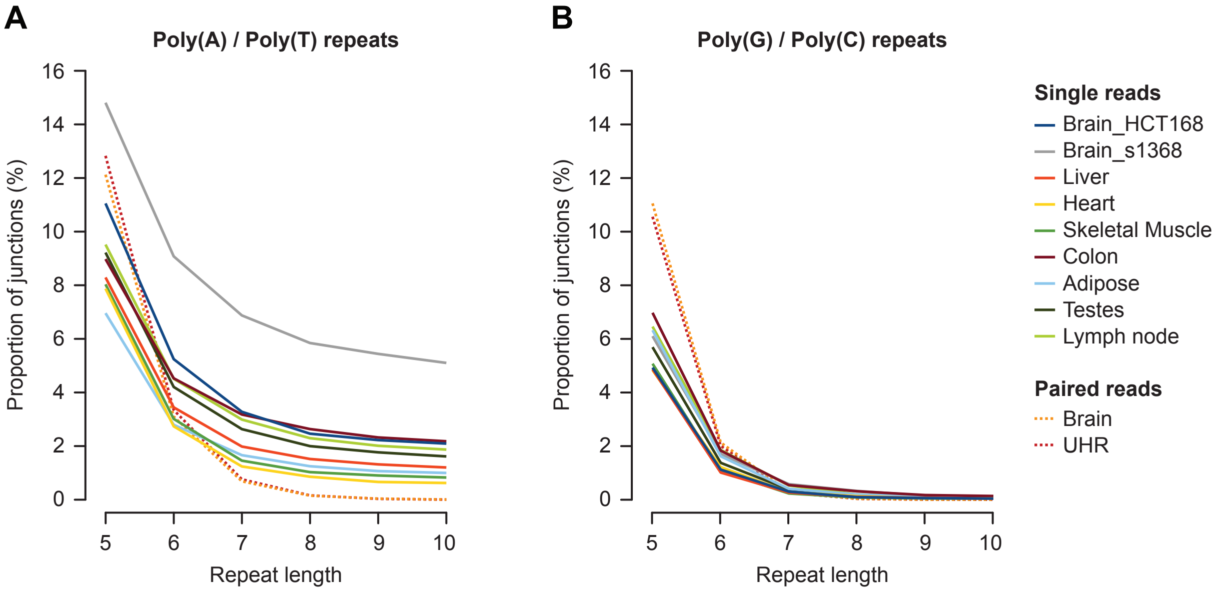

A
Poly(A) / Poly(T) repeats
16
14
12
10
8
6
4
2
0
Proportion of junctions (%)
5
6
7
8
9
10
Repeat length
B
Poly(G) / Poly(C) repeats
16
14
12
10
8
6
4
2
0
Proportion of junctions (%)
5
6
7
8
9
10
Repeat length
Single reads
Brain_HCT168
Brain_s1368
Liver
Heart
Skeletal Muscle
Colon
Adipose
Testes
Lymph node
Paired reads
Brain
UHR
